# Nutrient solutions for *Arabidopsis thaliana*: a study on nutrient solution composition in hydroponics systems

**DOI:** 10.1186/s13007-020-00606-4

**Published:** 2020-05-18

**Authors:** Sander H. van Delden, Mohammad Javad Nazarideljou, Leo F. M. Marcelis

**Affiliations:** 1 Horticulture and Product Physiology, Wageningen University, PO Box 16, 6700AA Wageningen, The Netherlands; 2 Department of Horticultural Science, Mahabad Branch, Islamic Azad University, Mahabad, Iran

**Keywords:** Electrical conductivity (EC), Nutrient solution, Salt (NaCl) response, Growth, Hydroponic system

## Abstract

**Background:**

There is little information on the effect of nutrient solutions composition on Arabidopsis growth. Therefore, we compared growth performance of *Arabidopsis thaliana* (Col-0) grown on the most commonly used nutrient solutions in deep water culture: Hoagland and Arnon, Murashige and Skoog, Tocquin, Hermans, and Conn. In addition to these nutrient solution composition experiments, we established Arabidopsis growth response curves for nutrient solution concentration and salt stress (NaCl).

**Results:**

Arabidopsis rosette fresh and dry weight showed an approximate linear decline with NaCl dose in deep water culture, i.e. 9% reduction relative to control per unit of electrical conductivity (EC in dS m^−1^, for scale comprehension 1 dS m^−1^ equals ~ 10 mM NaCl). The Tocquin, ½Hoagland and Conn nutrient solutions had equal and optimal growth performance. Optimal nutrient solution concentration for Tocquin and Hoagland was 0.8 to 0.9 dS m^−1^. Close to the EC of ½Hoagland (1.1 dS m^−1^), which is frequently used in Arabidopsis research. Conn solution showed optimal growth at much higher EC (2 dS m^−1^) indicating that it is a balanced nutrient solution that matches the needs of Arabidopsis. Full Murashige and Skoog solution (5.9 dS m^−1^) was lethal and diluted solutions (EC of 1.6 and 1.1 dS m^−1^) caused stress symptoms and severe growth retardation at later developmental stages.

**Conclusions:**

*Arabidopsis thaliana* (Col-0) plants grown in deep water culture showed a sixfold growth difference when commonly used nutrient solutions were compared. Murashige and Skoog solution should not be used as nutrient solution in deep water culture. Conn, Tocquin and ½Hoagland are balanced nutrient solutions which result in optimal Arabidopsis growth in hydroponic systems.

## Background

A short generation cycle, a completed genome sequence [1], numerous mutants, and high amenability to gene manipulation [2] make Arabidopsis (*Arabidopsis thaliana* L.) an important plant model [3]. Arabidopsis is mainly grown on soil or solid substrates. This type of cultivation can, however, hinder research related to plant nutrition. Water culture alleviates soil related problems such as root examination and impartial control over chemical substrate composition. In a solid substrate, it is often difficult to create micronutrient deficiencies. Moreover, temporal and spatial distribution of both pH and nutrients are difficult to assess and control. The growing number of studies in soilless systems indicates that using water culture is becoming more widespread [4–12]. There is, however, little information about the effect of nutrient solutions composition and nutrient solution concentration on Arabidopsis growth.

The electrical conductivity (EC in dS m^−1^) of a nutrient solution is proportional to the ion concentration of both macro- and micronutrients, i.e. EC indicates the amount of salts in solution. In water culture, EC is an important factor controlling growth, product quality and plant health [13–16]. Simply put, sub optimal EC levels lack a shoot growth stimulus and lead to nutrient deficiencies, while supra optimal EC levels reduce growth because of toxicities (osmotic and salt stress) [17]. The optimal EC of a nutrient solution can greatly differ between plant species [18–20] and has to the best of our knowledge not been established for Arabidopsis. Arteca and Arteca [9] is so far the only publication that reports the effects of nutrient solution concentration on biomass accumulation in hydroponically grown Arabidopsis. The nutrient solution tested by Arteca and Arteca [9] was developed by Somerville and Ogren [21] (Somerville solution from now on) and has hardly been used in Arabidopsis research, likely because Arteca and Arteca [9] reported lethal effects when EC exceeded 1.7 dS m^−1^ (Additional file 1 for the entire EC response). Looking at the growth of other species [13] one would expect that plants should be able to grow in full Somerville solution because an EC of 1.7 dS m^−1^ is relatively low. Therefore, the Arteca and Arteca [9] study on nutrient solution strength raises several questions: Is Somerville solution indeed lethal at an EC of 1.7 dS m^−1^? And if so, which component(s) of the nutrient solution formulation cause this poor performance? The only striking imbalance in the macronutrient composition is the high phosphate level (2.5 mmol L^−1^). Herefore, it would be interesting to do a full factorial study by growing Arabidopsis on relatively high phosphate level (2.5 mmol L^−1^) versus optimal (0.6 mmol L^−1^) in combination with either high (1.7 dS m^−1^) or optimal (0.7 dS m^−1^) EC.

The first objective of this paper is to compare the effect of commonly used nutrient solutions on plant performance (Exp. 1). Using a literature search protocol (Additional file 2) we selected the following five nutrient solutions for our main comparison: Hoagland and Arnon [22], Murashige and Skoog [23], Tocquin et al. [24], Hermans et al. [25] and Conn et al. [6]. To attain our first objective the selected nutrient solutions were used in a deep-water culture system and growth performance parameters were compared. In addition, the EC of the solutions was normalized to 1.1 dS m^−1^, to compare the effect of ion ratios, i.e. nutrient solution composition on Arabidopsis growth.

The second objective is to test if plants can grow on full Somerville solution and if high phosphate level is the main cause of poor nutrient solution performance (Exp. 2). To attain our second objective we used four Somerville solutions: plants were grown in full strength (1.7 dS m^−1^) or near optimal strength (0.7 dS m^−1^) and each strength (EC) was formulated using two phosphate (P) levels (0.6 and 2.5 mmol L^−1^). Hoagland solutions at P levels 0.6 and 2.5 mmol L^−1^ were used as reference.

The third objective is to use biomass accumulation and several other morpho-physiological traits to quantify salt stress sensitivity and the optimal nutrient solution concentration (Exp. 3). In order to quantify the sensitivity of Arabidopsis to salt stress in hydroculture, a salt (NaCl) dose response curve in the range of 0.5 to 32 dS m^−1^ was established. This was followed by high-resolution Hoagland nutrient solution experiments (Exps. 4 and 5) to quantify the optimal nutrient solution concentration in the range of EC 0.1 to 4.3 dS m^−1^.

## Results

### Effects of nutrient solution composition

**Fig. 1:**
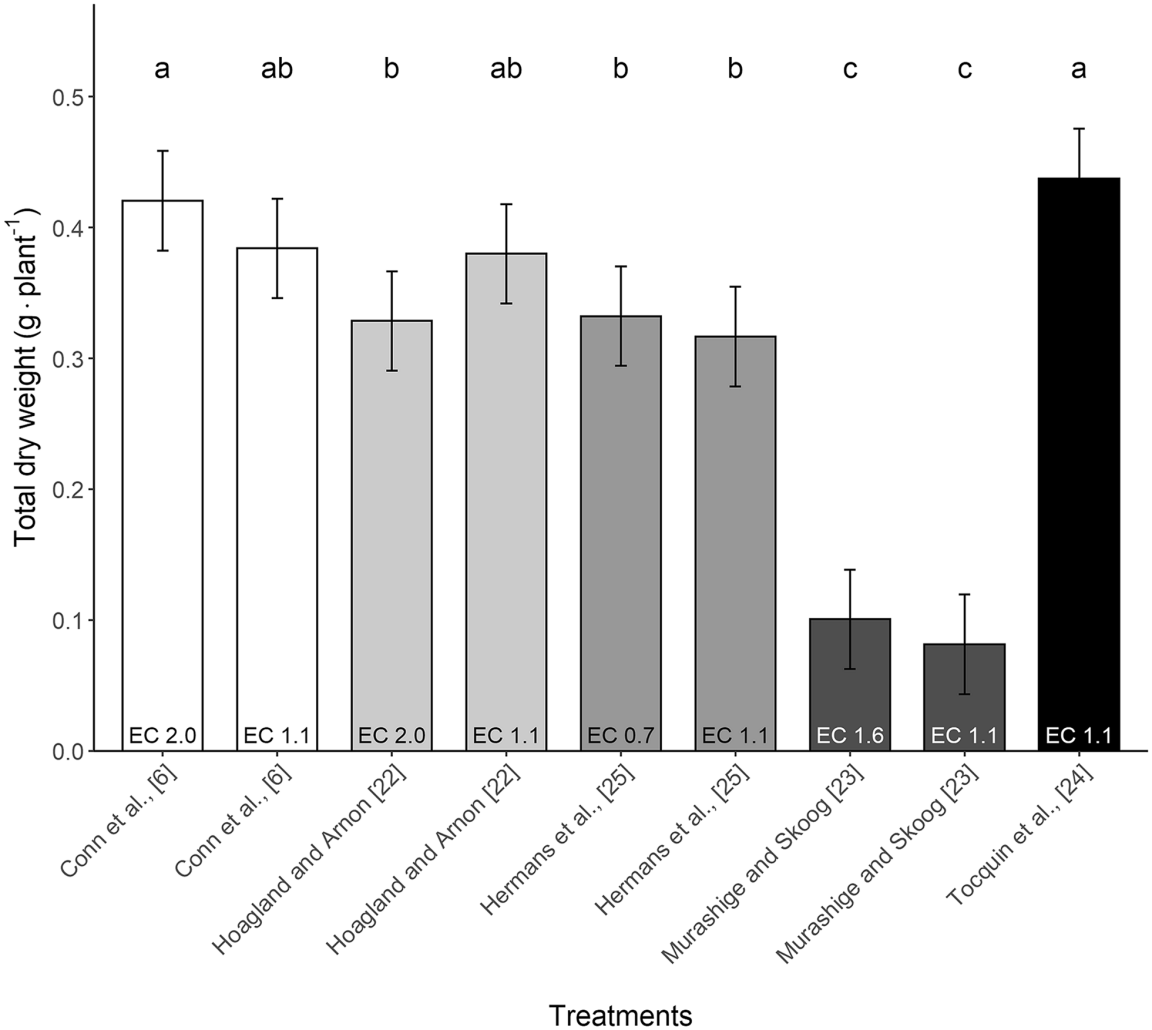
Effect of nutrient solution formulation and concentration (EC in dS m^−1^) on total dry biomass 48 days after sowing (DAS) of Arabidopsis Col-0 (Exp. 1). Bar size corresponds with the least square (LS) mean of a treatment and different colours identify different nutrient solutions. Error bar represents the 95% confidence interval of the LS mean (n = 6). LS means sharing the same letter are not significantly different (P > 0.05, Tukey-adjusted comparisons)

**Fig. 2:**
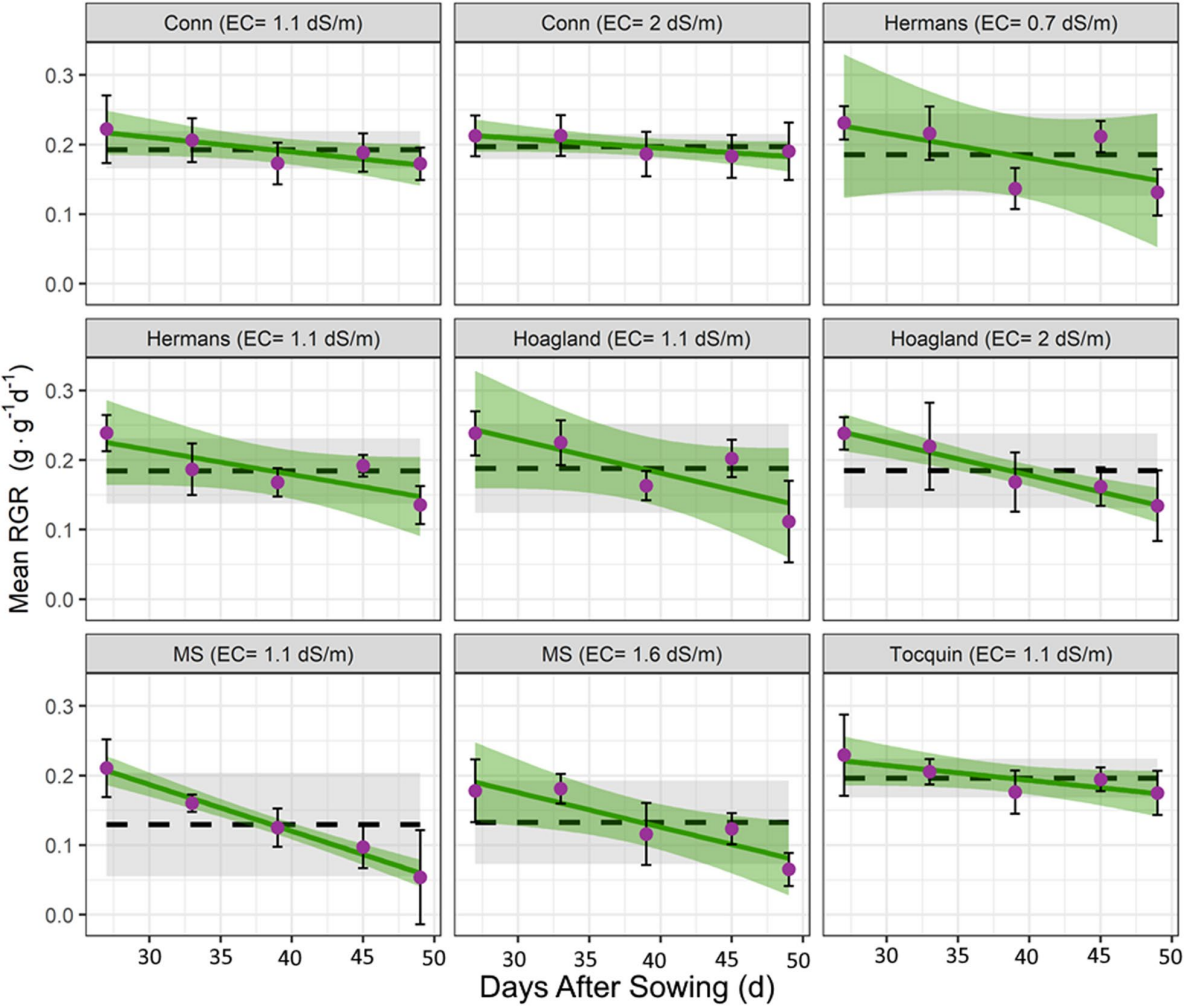
The mean relative growth rate (RGR) of Arabidopsis rosettes (circles) during 4 weeks grown in different nutrient solutions (Exp. 1). Error bar represents the standard deviation of the mean (n = 6). RGR data were fitted using either a constant relative growth rate (black dashed line, Eq. 1 with 95% confidence interval in grey) or a linearly declining relative growth rate (solid green line, Eq. 2 with confidence interval in green)

The five most commonly used nutrient solutions for Arabidopsis showed a sixfold difference in plant growth (Exp. 1, Fig. 1 and Table 1). Murashige and Skoog [23] (MS) nutrient solution had the poorest performance. The significantly lower yield became evident at 32 days after sowing (DAS) and onwards (Fig. 2 and Additional file 4). Among all tested solutions, the Tocquin [24] and Conn [6] solution resulted in the highest rosette dry weight 48 DAS (Table 1). Yet, the Hoagland [22] solution was not significantly different from the Tocquin and Conn solution when the Hoagland solution’s concentration was halved (Fig. 1, Table 1 and Additional file 3). The Hermans solution [25] resulted in lower biomass accumulation compared to the Tocquin solution (Table 1). When the concentration of the Hermans solution was increased, from EC 0.7 to 1.1 dS m^−1^ dry weight accumulation did not increase.

**Table 1:** Effect of nutrient solution on: rosette leaf fresh (LFW) and dry weight (LDW), dry matter percentage (DM), root dry weight (RDW), rosette:root ratio (R:R) at 48 days after sowing (DAS)

	EC (dS m^−1^)	LFW (g plant^−1^)	LDW (g plant^−1^)	DM (%)	RDW (g plant^−1^)	R:R
**Full nutrient solution**						
Conn et al. [6]	2.0	4.70 a	0.367 ab	7.82 c	0.0537 ab	6.9 a
Hoagland and Arnon [22]	2.0	3.40 b	0.286 c	8.50 c	0.0425 bc	7.6 a
Hermans et al. [25]	0.7	3.55 b	0.292 bc	8.21 c	0.0405 bc	7.5 a
Murashige and Skoog [23]	1.6	0.685 c	0.0687 d	10.2 a	0.0319 cd	2.7 c
Tocquin et al. [24]	1.1	4.83 a	0.375 a	7.85 c	0.0625 a	6.3 ab
**Normalized to EC 1.1 dS m**^**−1**^						
Conn et al. [6]	1.1	4.30 ab	0.333 abc	7.75 c	0.0513 ab	6.7 a
Hoagland and Arnon [22]	1.1	4.16 ab	0.334 abc	8.03 c	0.0460 abc	7.2 a
Hermans et al. [25]	1.1	3.21 b	0.278 c	8.88 bc	0.0386 bc	7.2 a
Murashige and Skoog [23]	1.1	0.643 c	0.0637 d	9.91 ab	0.0178 d	3.9 bc
Tocquin et al. [24]	1.1	4.83 a	0.375 a	7.85 c	0.0625 a	6.3 ab
**Standard error of the means**		0.247	0.0172	0.263	0.00415	0.6

Least square (LS) means (n = 6) sharing the same letter are not significantly different (P > 0.05, Tukey-adjusted comparisons)

The relative growth rate (RGR) of the Arabidopsis plants showed a linear decline (Fig. 2). To allow for a declining relative growth rate, the traditional growth equation for exponential growth was adapted, leading to an improved visual fit and goodness of fit parameters. In more detail, this led to an increase in ${\bar{\text{r}}}^{2}$ from 0.967 to 0.994 and AIC from − 1961 to − 2427 (Additional file 4 shows fitted curves). Comparing the effect of different nutrient solutions on the parameter estimates of Eqs. 1 and 2 revealed a similar trend. The best performing nutrient solutions had the highest RGR (Eq. 1) and the smallest decline in RGR (parameter “${\text{RGR}}_{\text{slope}}$”, Eq. 2) (see Additional file 4).

Root weight (Table 1) and root length (data not shown) of plants grown in MS solution were the lowest among all treatments. However, the rosette to root ratio was significantly lower for the MS solution compared to all other solutions (Table 1). In other words, root weight was relatively high compared to the low rosette weights of plants grown on MS solution.

Estimates of chlorophyll and nitrogen content on non-chlorotic part of the leaf showed significantly higher levels (~ 30%) in the Murashige and Skoog [23] solutions compared to all other solutions (Additional file 5 for bar graphs). This corresponds with its significantly higher dry matter content (Table 1). Notably, Murashige and Skoog [23] flavonoid and anthocyanin indices were not elevated (Additional file 5), but the plants exhibited clear stress symptoms, such as chlorosis (Additional file 6 for photos).

**Fig. 3:**
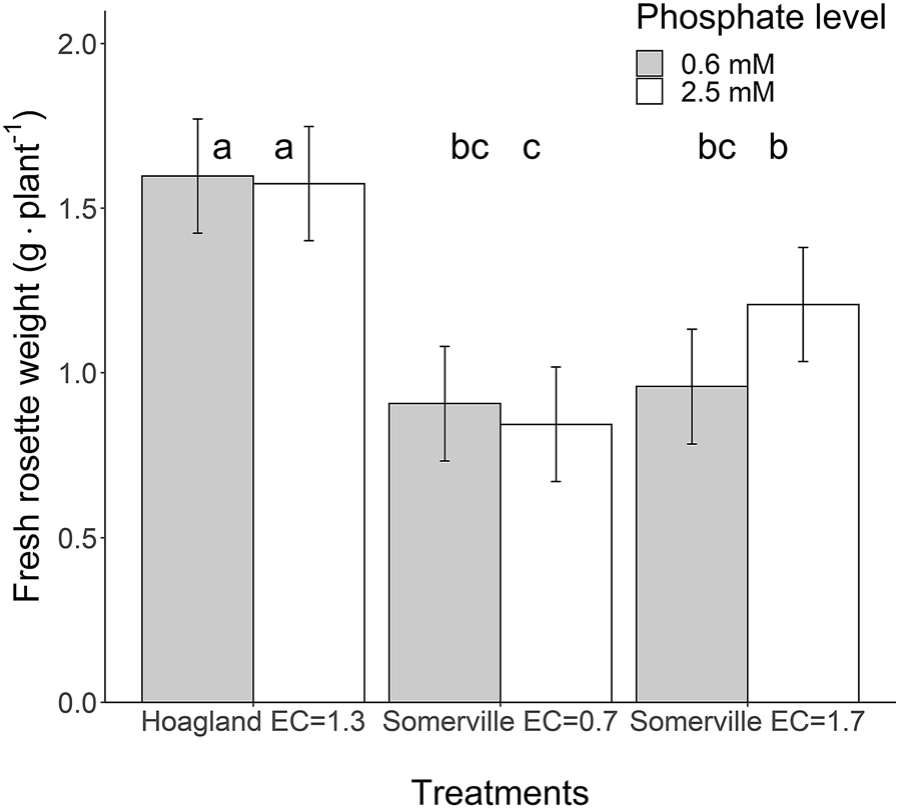
Effect of Somerville solution concentration (EC in dS m^−1^) and phosphate level on rosette fresh weight (g plant^−1^). Hoagland solution served as control (Exp. 2). Error bar represents the 95% confidence intervals of the least square (LS) means (n = 4 with each replicated represented by 3 plants). Means sharing the same letter are not significantly different (P > 0.05, Tukey adjusted comparisons)

In order to test if Somerville solution is indeed lethal at an EC of 1.7 dS m^−1^ [9] and whether relatively high phosphate level (2.5 mmol L^−1^) is the cause of this, six nutrient solutions were compared (Exp. 2). Somerville solution was tested in a full factorial design with high (1.7 dS m^−1)^ or optimal (0.7 dS m^−1)^ EC at two phosphate levels: either high (2.5 mmol L^−1^) or optimal phosphate (0.6 mmol L^−1^) level. A frequently used Hoagland solution (1.3 dS m^−1)^ with either high (2.5 mmol L^−1^) or optimal phosphate (0.6 mmol L^−1^) was used as control treatment. We found that the relatively high phosphate levels (2.5 mmol L^−1^) were not the main cause of the Somerville nutrient solution’s poor performance (Fig. 3). Lowering the phosphate level from 2.5 to 0.6 mmol L^−1^ did not affect growth (P = 0.43). However, overall growth performance of the Somerville solution was significantly lower compared to the Hoagland solution. Surprisingly, the “full strength (1×)” Somerville solution with an EC of 1.7 dS m^−1^, which was lethal in the Arteca and Arteca [9] study, performed better than the diluted solution with EC of 0.7 dS m^−1^ (P = 0.004, Fig. 3), which was the optimal EC in the Arteca and Arteca [9] study.

### Effects of nutrient solution concentration

**Fig. 4:**
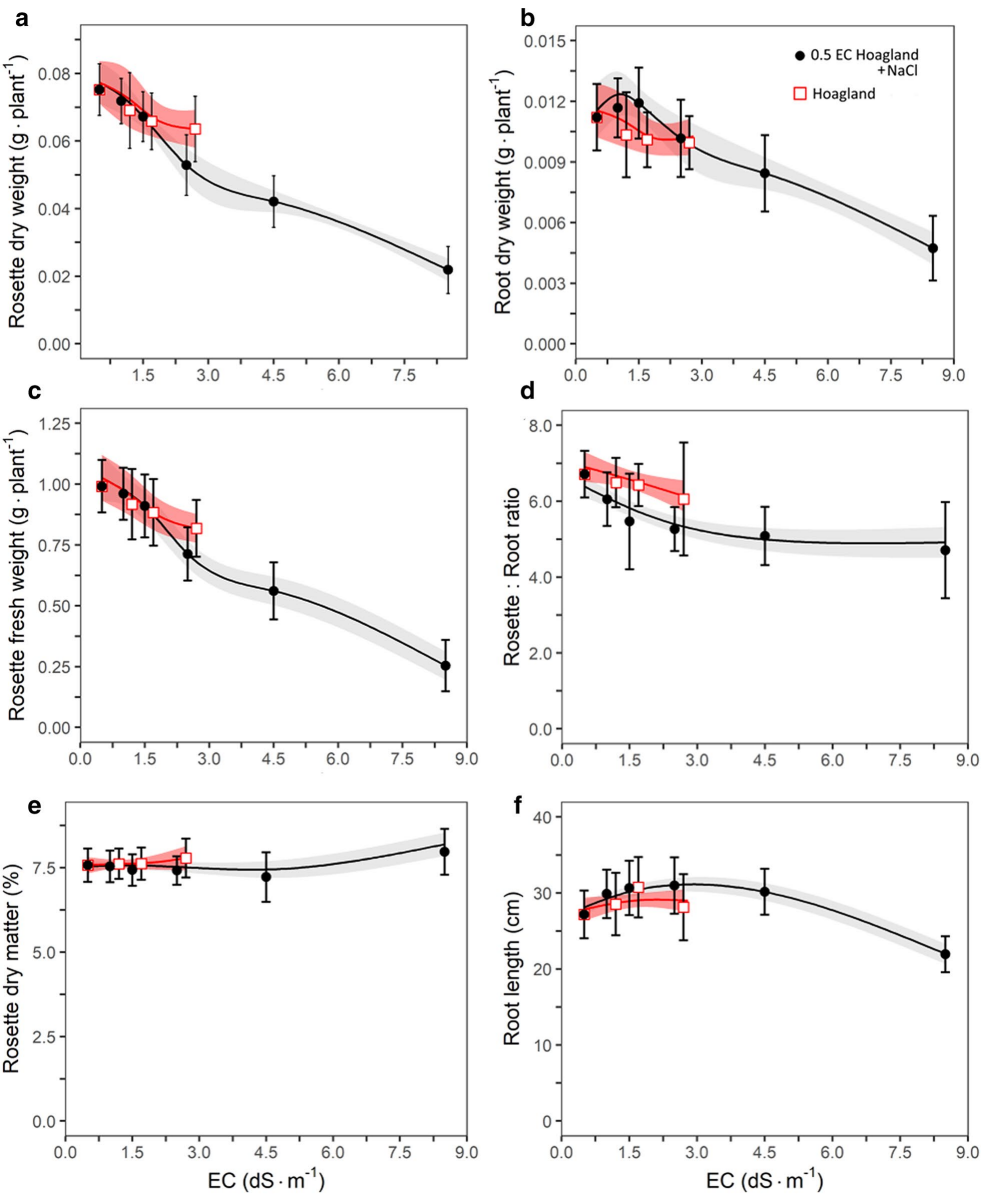
Effects of NaCl and Hoagland nutrient solution concentration (EC in dS m^−1^) at 38 days after sowing (DAS) on: **a** rosette dry weight, **b** root dry weight, **c** rosette fresh weight, **d** rosette:root ratio, **e** dry matter % and **f** root length (n = 6) (Exp. 3). Red (Hoagland) and grey (NaCl) bands represent the confidence interval of the spline function (red and black lines) fitted to the data. Symbols represent the means and are averages of 6 true replicates and each replicate is represented by 4 plants, i.e. 24 data points per mean. Error bars represent the standard deviation of the mean. Data points of EC 16 and 32 dS m^−1^ are excluded as plants died during early development

**Fig. 5:**
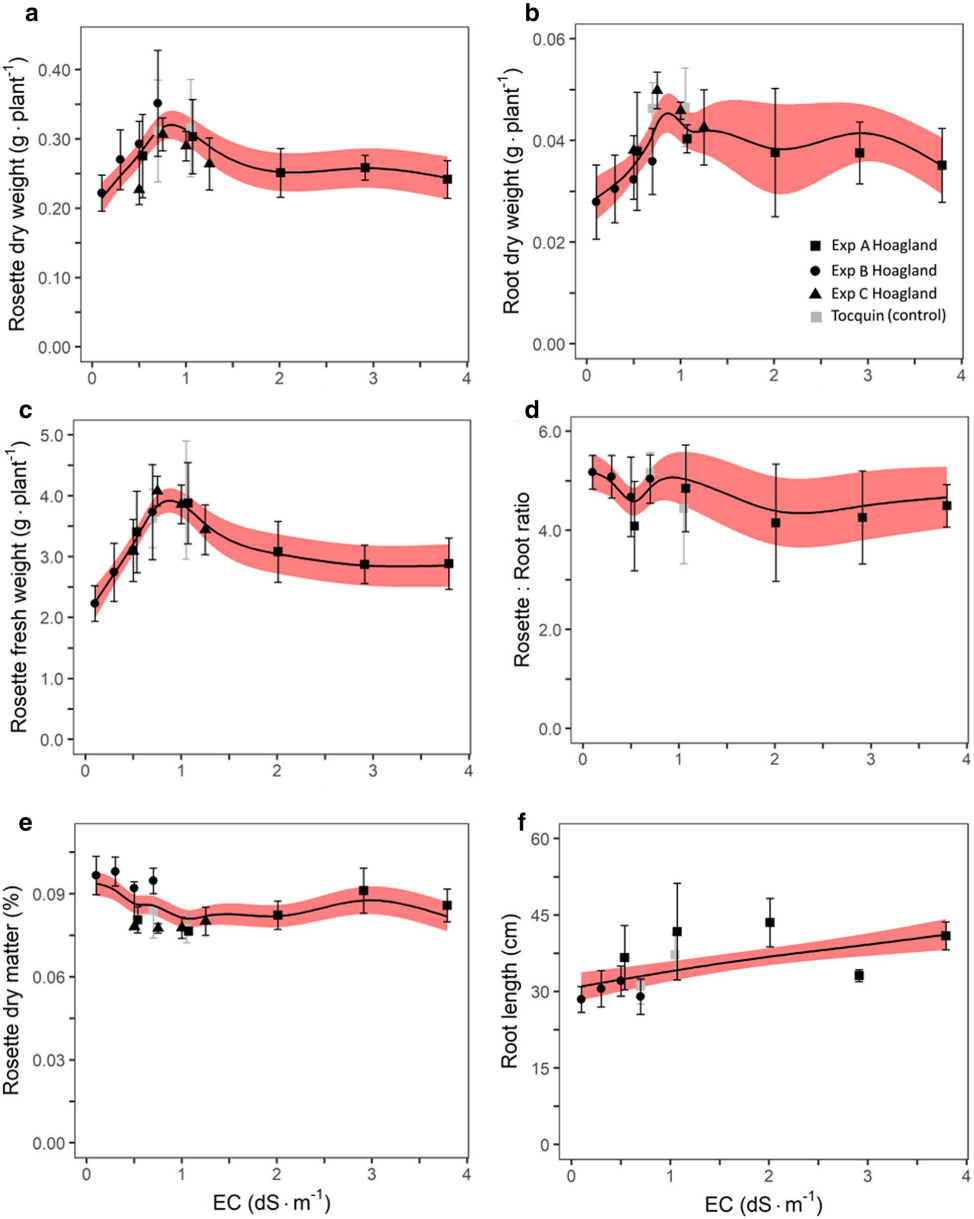
Effects of Hoagland nutrient solution concentration (EC in dS m^−1^) on **a** rosette dry weight, **b** root dry weight, **c** rosette fresh weight, **d** rosette:root ratio, **e** dry matter % and **f** root length at 48 days after sowing (DAS) (Exp. 4 and 5). Symbols represent the mean (6 ≤ n ≤ 10) error bars represent the standard deviation of the mean. The black line is a modelled spline to show the data pattern with its confidence interval in transparent red. Distinct model optima are **a** 0.37 g at EC 0.84 dS m^−1^**b** 0.046 g at EC 0.86 dS m^−1^**c** 3.88 g at EC 0.88 dS m^−1^

Testing salt sensitivity by varying NaCl level showed that Arabidopsis was salt stress sensitive (Exp. 3, Fig. 4). In line with its sensitivity to salts stress, we found that optimal macronutrient concentration of Arabidopsis is relatively low compared to other plants, i.e. in the range of 0.5 and 1.25 dS m^−1^ (Exps. 4 and 5, Fig. 5). Although spline curves were fitted to the data of the NaCl dose response curves to detect possible patterns (Fig. 4), rosette biomass showed and approximately linear decline with increasing NaCl concentration. When fitting a linear model, the slope for fresh weight was 0.098 g dS m^−1^ with an intercept of 1.05 g, i.e. 9.3% reduction per dS m^−1^. The fitted slope for dry weight was 0.0071 dS m^−1^ with intercept 0.079 g, i.e. 9.0% reduction per dS m^−1^. The slope of this decline was not significantly different between the NaCl solution and Hoagland solutions in either fresh (P = 0.94) or dry (P = 0.68) weight. The fitted spline indicated an initial increase in root dry weight at the lower three NaCl concentrations, but these three treatments did not significantly differ (P = 0.41). Rosette:root ratio, however, was systematically lower for the NaCl treatment compared to the Hoagland solution. The decline of leaf area with increasing NaCl dose followed a similar trend as the biomass decline (Additional file 7).

To estimate the optimal EC for Arabidopsis growth with high precision we used Hoagland solution, as Hoagland solution is the most frequently used nutrient solution for Arabidopsis deep water culture (Fig. 5). The EC dose-biomass accumulation response showed a clear optimal range between 0.8 to 0.9 dS m^−1^ (Fig. 5). Tocquin nutrient solutions at EC 0.7 and 1.1 dS m^−1^ were used as control and corresponded well with the Hoagland solution plant response curve (Fig. 5).

In the optimal EC range (0.5 to 1.25 dS m^−1^) there were no significant differences in either maximum quantum yield (Fv/Fm) or photosystem II efficiency ($\Phi_{\text{PSII}}$). All values for both parameters were in the range of unstressed plants (0.70–0.85). Counted through the entire growth period, any differences in the number of leaves between Hoagland concentrations were not substantial. However, around 45 DAS there was a small but significantly higher number of leaves at both EC 0.7 dS m^−1^ (mean 36.8, sd 0.98 leaves) and 1.1 dS m^−1^ (mean 35.7, sd 1.8 leaves) compared to EC 0.5 dS m^−1^ (mean 33.1, sd 1.8 leaves) and EC 1.25 dS m^−1^ (mean 33.4, sd 1.13 leaves) (Additional file 8).

Care was taken to maintain a stable pH during the nutrient solution composition experiments by using a large container size (20 L), weekly nutrient solution refreshments and the addition of MES buffer (0.25 g L^−1^). In our pilot studies preceding the nutrient solution concentration experiments, unbuffered Tocquin solution in smaller 2 L trays was stable until 37 DAS, but pH increased rapidly during the last weeks of cultivation. Therefore we tested several NH_4_^+^:NO_3_^−^ ratios: 0.03, 0.05 and 0.07; with and without 0.5 mmol HCO_3_ as buffer. The higher NH_4_^+^:NO_3_^−^ ratio in Hoagland solution (0.07) created a more stable solution than the lower ratio of 0.02 in Tocquin solution (Additional file 9). Adding 0.5 mmol L^−1^ HCO_3_ as a buffer did not results in a more stable pH but did raise the nutrient solution pH to ~ 6.2 (Additional file 9 provides examples of pH dynamics).

## Discussion

*Arabidopsis thaliana* (Col-0) plants grown in deep water culture showed a sixfold growth difference when commonly used nutrient solutions were compared. The best performing nutrient solutions were Conn [6], Tocquin [24] and ½Hoagland [22], and the poorest performing solution was Murashige and Skoog [23] (MS) (Fig. 1 and Table 1). Using MS as nutrient solution in deep water culture resulted in plants with several stress symptoms and severe growth retardation at later developmental stages (Table 1, Fig. 1). The significantly lower yield caused by MS solution usage became evident at 32 DAS and onwards (Fig. 2 and Additional file 4). Estimates of chlorophyll and nitrogen content showed significantly higher levels (~ 30%) in the MS solutions (Additional file 5). This corresponds with their elevated dry matter content and stagnant fresh weight accumulation. MS solution has relatively high potassium (K = 20 mmol L^−1^) and nitrogen levels (NH_4_^+^ = 20 and NO_3_^−^ = 39 mmol L^−1^) with a high NH_4_^+^:NO_3_^−^ ratio (0.52). High NH_4_^+^ concentration generally causes toxicity in plants [26]. Due to the high K and N levels and relatively low phosphate concentration (P = 1.25 mmol L^−1)^ the N:P ratio is very high (47.2), as are the ratio between the cations K:Ca (6.7) and K:Mg (13.4) (Table 3). All together, the MS solution is completely imbalanced and is not suitable for optimal growth in deep water culture.

Between 2015 and 2018, we found that 90 studies used a hydroponic system for Arabidopsis research, 23 out of 90 used MS medium as a nutrient solution (Additional file 2). The recently published study of Nathoo et al. [27] is a good example of the suboptimal growth caused by MS medium. Pictures of the cultivated plants in the publication clearly show a stressed phenotype; any treatment applied, or gene expression analysis performed on these plants will be confounded by these elevated stress levels.

Among all tested solutions, Tocquin and Conn solution resulted in the highest rosette leaf dry weight 48 days after sowing (DAS) (Table 1). Yet, when the Hoagland solution concentration was halved (1.1 dS m^−1^) the Hoagland solution did not perform significantly different than the Tocquin and Conn solution (Fig. 1, Table 1 and Additional file 3). The Hermans solution [25] resulted in lower biomass accumulation compared to Tocquin and Conn (Table 1). When the concentration of the Hermans solution was increased, from EC 0.7 to 1.1 dS m^−1^ dry weight accumulation did not increase.

The optimal nutrient solution concentration for Tocquin and Hoagland lies within the EC range of 0.8 to 0.9 dS m^−1^ (Fig. 3). Yet, Conn nutrient solution still showed good performance at an EC of 2 dS m^−1^ and lowering the EC of Conn solution to 1.1 dS m^−1^ did not significantly change the growth response. This indicates that Conn nutrient solution has the best balance between macronutrients of the solutions tested in this study.

The pH stability of the Tocquin solution was lower than that of Hoagland. At later growth stages, supplementing NH_4_^+^ maintains a stable NH_4_^+^:NO_3_^−^ ratio that will counteract a potential pH increase. Note that high NH_4_^+^ levels at the beginning of the cultivation period might acidify the nutrient solution too much depending on the microbiome present in the cultivation system [28]. Because we used MES buffer in the nutrient solution comparisons trials, the pH stability of unbuffered Conn solution was unfortunately not tested. However, based on the NH_4_^+^:NO_3_^−^ ratio and a pilot experiment in which unbuffered Conn solution was used, the pH of Conn solution without MES buffer is likely to be rather stable.

The NaCl response curve (Fig. 4) of biomass accumulation is very similar to the one reported in the review of Munns and Tester [29], who showed a curve that was extrapolated based on three data points from Cramer [30]. As pointed out by Munns and Tester [29], Arabidopsis is relatively salt stress sensitive, which is in line with our findings (Fig. 4), and it has a relatively low optimal EC (Fig. 5). Yet, Conn nutrient solution does contain relatively high amounts of sodium (Na = 1.6 mmol L^−1^) and chlorine (Cl = 3.7 mmol L^−1^) compared to other solutions where Na and Cl amount were much lower (Table 3 and Additional file 10 for visual comparison). However, it is known from agronomic research that moderate amounts of sodium and chlorine can be beneficial for plant growth [31, 32].

To the best of our knowledge, this is the first research that compares many nutrient solution formulations and concentrations. Some publications mention a nutrient solution optimisation procedure in their lab, but do not report detailed results of these endeavours [6, 24]. Arteca and Arteca [9] is the only publication that reports the effects of the nutrient solution concentration on biomass accumulation in hydroponically grown Arabidopsis. The nutrient solution tested by Arteca and Arteca [9] was developed by Somerville and Ogren [21] (Somerville solution) and has hardly been used in Arabidopsis research, probably because its reported lethal effect at a relatively low upper threshold of ion concentration (1.7 dS m^−1^). Our study showed that the high phosphate levels were not the main cause of the poor performance of Somerville nutrient solution, since lowering the phosphate level from 2.5 to 0.6 mmol L^−1^ did not affect growth (P = 0.43, Fig. 3). Overall growth performance of the Somerville solution was indeed significantly lower compared to ½Hoagland solution. Unexpectedly, the proclaimed lethal full strength (1×) Somerville solution with an EC of 1.7 dS m^−1^ performed better than the “optimal ¼ concentration solution” EC of 0.7 dS m^−1^ (P = 0.004, Fig. 3). Arteca and Arteca [9] reported severe epinasty, i.e. curled and deformed leaves, when using 1× solution strength. We hypothesize that the contact between the sponges and the nutrient solution used in the Arteca and Arteca [9] system caused salt stress, especially at higher EC. When the nutrient solution in our growth system was left in contact with the agar plugs during the entire growth period, especially at higher EC levels, we also observed severe salt stress symptoms with epinastic, i.e. curled leaves (Additional file 11 for photos). The high evaporation rate caused high salt concentration on top of the agar plug. Lowering the water level to 1 or 2 cm below the plug, after the roots fully penetrated the agar plugs and came in contact with the solution, completely abolished all visible salt stress symptoms. As documented in detail in Nazarideljou et al. [33] other cultivation system optimizations that significantly improved plant growth were: size reduction of the microtube that holds the plant from 2.5 to 1 cm length, creating a high humidity in the mini greenhouses (> 90%) at seedling stage, and a larger growth space than the pipet trays that are traditionally used at early stages of development [6].

HYPONeX (Japan Co Ltd) (Tables 3 and 4) is a commercially available nutrient solution used in Arabidopsis research [34–38]. Although this solution performs well in substrate-based systems, it is lethal when we used it as nutrient solution in deep water culture. The high NH_4_^+^:NO_3_^−^ ratio of HYPONeX caused strong acidification of the nutrient solution in deep water culture. In stonewool and peat based systems the pH decrease caused by HYPONeX use is most likely counterbalanced by the “natural” pH increase caused by algae [39]. Peat soils possess, moreover, a cation ion exchange complex that the NH_4_^+^ molecules can adhere to, lowering the concentration that is in direct contact with the roots.

Several nutrient solutions used for Arabidopsis, including MS, Tocquin and Somerville contain cobalt (Co). Cobalt is not essential for plant growth. It induces oxidative stress [40], however, there could be some beneficial effects of leaf senescence retardation through inhibition of ethylene biosynthesis as reviewed by Pilon-Smits et al. [32]. As supported by our research, plants grow well without Cobalt, which is exemplified by Conn and Hoagland solution. Cobalt is, however, essential for N_2_ fixing plants [41] because it is part of the coenzyme cobalamin (vitamin B_12_) which is important in nodule metabolism [42, 43].

In this study, we used climatic conditions which are representative of settings often used in Arabidopsis research [44]. However, note that climatic conditions such as temperature and irradiance can influence plant composition and physiology and thereby influence the requirements of the nutrient solution for optimal growth. Thus, when using sub or supra optimal temperatures or light conditions effects of nutrient solutions might be different, and adjustments could be required.

In conclusion, Murashige and Skoog [23] solution should not be used in deep water culture as it results in severe growth retardation and stressed plants. Conn [6], Tocquin [24] and ½Hoagland [22] solution showed comparable optimal growth performance (Table 1). Optimal nutrient solution concentration for Tocquin and Hoagland lies in the EC range of 0.8 to 0.9 dS m^−1^ (Fig. 5). This is close to the EC of ½Hoagland (1.1 dS m^−1^), which is frequently used in Arabidopsis research. Conn solution maintained optimal growth at a relatively high EC (2 dS m^−1^), indicating that Conn is a balanced nutrient solution that matches the needs of Arabidopsis. Arabidopsis growth shows an approximate linear decline with NaCl dose in deep water culture (− 0.098 g dS^−1^ m for fresh weight and − 0.0071 g dS^−1^ m for dry weight). To prevent toxic high salt concentration on top of the plant holding agar plugs, water level should be 1 or 2 cm below the agar plug after the seedling roots have fully penetrated these plugs.

## Methods

Five experiments were performed as summarized in Table 2.

**Table 2:** Short description of each conducted experiment together with their abbreviation

Experiment abbreviation	Description
Exp. 1	Evaluation of five nutrient solutions: Conn [6], Hoagland [22], Hermans [25], Murashige and Skoog (MS) [23], and Tocquin [24]
Exp. 2	Comparison of Somerville solution [9, 21] at different electrical conductivity (EC) and phosphate levels using Hoagland [22] as control treatment
Exp. 3	A salt (NaCl) dose response experiment in the range of 0.5 to 32 dS m^−1^ to establish the response of Arabidopsis to salt (ionic) stress in deep water culture
Exp. 4	The effects of Hoagland [22] nutrient solution concentration in the range of 0.1 to 0.7 dS m^−1^ including Tocquin [24] solution as reference/control
Exp. 5	The effects of Hoagland [22] nutrient solution concentration in the range of 0.5 to 1.25 dS m^−1^ including Tocquin [24] solution as reference/control

### Environmental conditions

All experiments were carried out in fully controlled climate rooms of Wageningen University, Wageningen, the Netherlands. Climate room conditions were set to 16:8 h light/dark cycle with 70% atmospheric humidity at 22/20 °C day/night temperature [6]. The average light intensity at the plant level (rosette) was maintained at 175 µmol m^−2^ s^−1^ supplied by fluorescent tubes (Phillips TL-D 58 W/40). CO_2_ levels were kept at ambient levels, approximately 400 ppm.

### Plant material, germination and seedling growth

Wild type Arabidopsis seeds (*Arabidopsis thaliana* ecotype Columbia L Heynh (Col-0)) were surface-sterilized by immersion in ethanol 70% (w/w) for 5 min and afterwards were rinsed three times with sterile deionized water in a flow cabinet. To synchronize germination, seeds were placed on autoclaved wet filter paper in a sterile petri dish and were stored 72 h at 4 °C in darkness [44]. Polypropylene microtubes (0.5 mL, Sarstedt^®^ reference 72.698.200) were cut to 1 cm below the rim. This 1 cm tube length was carefully chosen after an optimisation experiment testing longer and shorter tubes [33]. The tubes were autoclaved and fixed upside down on adhesive brown scotch tape in a laminar flow cabinet. Subsequently, the tubes were filled with 0.3 mL of germination medium using an Eppendorf Multipette^®^ M4. The germination medium consisted of 50% Daishing agar (2.2% W/V) and 50% full Hoagland and Arnon 2 (1950) nutrient solution (Hoagland solution from now on) resulting in a solid medium with half strength Hoagland solution. The Daishing agar (CAS number 9002-18-0) and the full Hoagland solution were sterilized by autoclaving and filter sterilization (Whatman™ 25 mm GD/X) respectively. To avoid solidification of the medium while filling the microtubes the germination medium was kept at 70 °C with constant stirring using a magnetic stirrer and hot plate. After the agar solidified, the microtubes were placed in Greiner bio one filter tip “blue boxes” (Additional file 12 for picture) 11 × 7.6 × 7.3 cm with 1 cm space between the microtube tube holders and the lid). The blue boxes were filled with 452 mL of half strength Hoagland nutrient solution. One seed per microtube was placed superficially in the agar and the lid of the blue box was closed. The box was placed in the climate room and seedlings were gradually exposed to the climate room conditions. This was done 4 days after sowing (DAS) by lifting one side of each lid of the blue boxes, 1 day later the other side of the lid was opened, i.e. leaving the lid on top of the box, but not adhered to it. Six DAS the lid was removed, and seedlings were grown in the blue boxes for an additional 10 days before exposure to the nutrient solution treatments.

To acclimatize the seedlings to new nutrient solution recipes (Exp. 1), 14 DAS one-third of the nutrient solution in seedling containers was replaced with a new nutrient solution. Half of the existing solution was exchanged with a new recipe on 15 DAS, and the entire solution was replaced with treatment solutions on 16 DAS [6]. For the nutrient solution composition trials (Exp. 1) at 20 DAS, 11 equal sized plants were transferred into aerated polypropylene (PP) UTZ^®^ containers, filled with approximately 20 L nutrient solution of each treatment. Plants were positioned such that they were not touching during the entire growth period. Nutrient solutions of Hoagland and Arnon [22], Murashige and Skoog [23], Tocquin [24], Hermans [25], and Conn [6] were all prepared in both full strength and normalised to EC 1.05 dS m^−1^ (Tables 3 and 4); the pH was set to 5.6 by applying 0.5 M KOH (base) or H_2_SO_4_ (acid) and stabilized using MES buffer (0.25 g L^−1^).

**Table 3:** Macronutrient solution composition of several nutrient solutions and their dilutions with corresponding electronic conductivity (EC)

	EC (dS m^−1^)	Macronutrients (mmol L^−1^)										
		NO_3_	NH_4_	P	K	Ca	Mg	SO_4_	Na	Cl	NH_4_:NO_3_	K:Ca
**Full nutrient solution**												
Hermans et al. [25]	0.7	2.00	0.00	0.25	2.01	1.00	1.00	0.00	1.88	0.00	0.00	2.01
Murashige and Skoog [23]	5.9	39.40	20.60	1.25	20.06	3.00	1.50	1.73	0.40	6.00	0.52	6.69
Murashige and Skoog [23] ($${\raise0.7ex\hbox{$1$} \!\mathord{\left/ {\vphantom {1 4}}\right.\kern-0pt} \!\lower0.7ex\hbox{$4$}} \times$$)	1.6	9.85	5.15	0.31	5.03	0.75	0.38	0.43	0.10	1.50	0.52	6.71
Conn et al. [6]	2.0	9.00	2.00	0.60	5.60	2.10	2.00	2.02	1.60	3.70	0.22	2.67
Hoagland and Arnon [22]	2.0	14.00	1.00	1.00	6.00	4.00	2.00	2.00	0.00	0.02	0.07	1.50
Tocquin et al. [24]	1.1	7.15	0.16	0.13	5.10	1.01	0.50	0.52	0.03	0.00	0.02	5.05
HYPONeX Japan Co. Ltd.	1.4	4.40	1.70	1.30	4.10	2.00	1.20	3.30	0.10	0.00	0.39	2.05
Somerville and Ogren [21] used by Arteca and Arteca [9]	1.6	9.00	0.00	2.50	7.50	2.00	2.00	2.02	0.03	0.00	0.00	3.75
**Normalized to EC 1.1 dS m**^**−1**^												
Conn et al. [6]	1.1	4.68	1.04	0.31	2.91	1.09	1.04	0.83	1.05	1.92	0.22	2.67
Hoagland and Arnon [22]	1.1	7.00	0.50	0.50	3.00	2.00	1.00	1.00	0.00	0.02	0.07	1.50
Hermans et al. [25]	1.1	3.22	0.00	0.40	3.23	1.61	1.61	3.03	0.00	0.00	0.00	2.01
Murashige and Skoog [23]	1.1	6.45	3.37	0.20	3.28	0.49	0.25	0.28	0.07	0.98	0.52	6.69
**Dilutions**												
Hoagland and Arnon [22]	0.1	0.57	0.04	0.04	0.24	0.16	0.08	0.08	0.00	0.02	0.07	1.50
Hoagland and Arnon [22]	0.3	1.76	0.13	0.13	0.75	0.50	0.25	0.25	0.00	0.02	0.07	1.50
Hoagland and Arnon [22]	0.5	3.00	0.21	0.21	1.29	0.86	0.43	0.43	0.00	0.02	0.07	1.50
Hoagland and Arnon [22]	0.7	4.27	0.31	0.31	1.83	1.22	0.61	0.61	0.00	0.02	0.07	1.50
Tocquin et al. [24]	0.7	4.69	0.10	0.09	3.34	0.66	0.33	0.34	0.03	0.00	0.02	5.06
Hoagland and Arnon [22]	1.0	6.24	0.45	0.45	2.68	1.78	0.89	0.89	0.00	0.02	0.07	1.51
Hoagland and Arnon [22]	1.3	7.93	0.57	0.57	3.39	2.27	1.13	1.13	0.00	0.02	0.07	1.49
Hoagland and Arnon [22]	3.0	21.00	1.50	1.50	9.00	6.00	3.00	3.00	0.00	0.02	0.07	1.50
Hoagland and Arnon [22]	3.8	28.00	2.00	2.00	12.00	8.00	4.00	4.00	0.00	0.02	0.07	1.50
**Phosphate experiment**												
Hoagland and Arnon [22]	1.4	7.00	0.50	0.50	5.00	2.00	1.00	2.00	0.00	0.02	0.07	2.50
Hoagland and Arnon [22]	1.3	7.00	0.50	2.50	5.00	2.00	1.00	1.00	0.00	0.02	0.07	2.50
Somerville and Ogren [21]	1.7	9.00	0.00	2.50	7.50	2.00	2.00	2.00	0.17	0.01	0.00	3.75
Somerville and Ogren [21]	1.8	9.00	0.00	0.63	7.50	2.00	2.00	2.94	0.17	0.01	0.00	3.75
Somerville and Ogren [21]	0.7	2.25	0.00	2.50	3.75	0.50	0.50	0.50	0.17	0.01	0.00	7.50
Somerville and Ogren [21]	0.7	2.25	0.00	0.63	3.75	0.50	0.50	1.44	0.17	0.01	0.00	7.50

Salt recipes are provided in Additional file 13

**Table 4:** Micro element composition of all nutrient solutions used

Nutrient solution	Micronutrients (mmol L^−1^)							
	Fe	B	Cu	Zn	Mn	Mo	I	Co
Hermans et al. [25]	20.0	10.0	0.10	1.00	1.00	0.07	0.00	0.00
Murashige and Skoog [23]	100	100	0.32	30.0	100.0	1.00	5.00	0.10
Murashige and Skoog [23] ($${\raise0.7ex\hbox{$1$} \!\mathord{\left/ {\vphantom {1 4}}\right.\kern-0pt} \!\lower0.7ex\hbox{$4$}} \times$$)	25.0	25.0	0.08	7.50	25.0	0.25	1.25	0.03
Conn et al. [6]	50.0	50.0	0.50	10.0	5.00	0.10	0.00	0.00
Hoagland and Arnon [22]	40.0	46.3	0.32	0.77	9.15	0.11	0.00	0.00
Tocquin et al. [24]	22.4	9.68	0.22	0.31	2.03	0.14	0.00	0.09
HYPONeX Japan Co. Ltd.	50.0	23.0	0.30	0.66	4.50	0.13	0.00	0.00
Somerville and Ogren [21] used by Arteca and Arteca [9]	17.0	70.0	0.50	1.00	14.0	0.20	0.00	0.01

Salt recipes are provided in Additional file 13

During the seedling stage (< 20 DAS) of the nutrient solution experiment (Exp. 1) seedling growth was not uniform. This problem was initially solved by restarting the experiment and sowing extra seedlings to obtain enough healthy and equal sized plants. However, for all other experiments we optimized the seedling growth protocol as published in Nazarideljou et al. [33]. In this optimized protocol, which was used in Exps. 2–5, seed pre-treatments and preparation of the microtubes with agar was similar to the nutrient solution trial (Exp. 1). However, in this new protocol we used mini greenhouses (air volume of 8 L), containing two black 0.7 L polypropylene (PP) nutrient solution tanks (18.2 × 13.5 × 4.5 cm) that were each covered with a BPA-free PVC lid (20 × 15 cm) and contained 35 holes for microtubes (Additional file 12). The bottom trays of the mini-greenhouses itself contained 100 mL tap water to increase humidity in the mini greenhouse. Contact was ensured between the nutrient solution with the lower part of the tube to avoid cracking or germination medium sliding through the microtube tube. Additionally, the microtubes were fully filled to prevent air bubbles at the bottom of the tube, which would have hindered contact between the agar and the nutrient solution (Additional file 12). After 7 days the ventilation windows of the mini-greenhouses covers were gradually (3 day period) opened, 11 DAS the mini-greenhouse covers were removed.

For the NaCl (Exp. 3) and Hoagland concentration experiments (Exps. 4 and 5), 20 DAS plant were transferred to 2 L containers (18 × 13 × 13 cm) that contained the treatment solutions. Dilution factor for each solution was accurately calculated using a R-script (Additional file 14) that was based on the Truesdell-Jones ion activity model as explained at http://aqion.de. EC, pH and dissolved oxygen where measured with a calibrated Orion Star™ A329 pH/ISE/Conductivity/Dissolved Oxygen sensor after the nutrient solutions were prepared. pH was set to 5.6 but MES buffer was not used in these trails.

In all experiments, nutrient solutions were aerated such that dissolved oxygen content did not drop below 90% saturation. Aeration was only required at final growth stages. Air bubbles were slowly released from the tube to perturbate the water slightly and break the surface tension to improve oxygen diffusion into the water. High root perturbation resulted in growth retardation and yellowing of leaves. Nutrient solution pH, EC and percentage of dissolved oxygen were measured at least three times a week, and, when setpoint deviations were observed, they were checked daily. Additionally, the ion concentrations, EC and pH of all nutrient solution formulations were tested before usage by a certified nutrient solution testing company (Eurofins Agroscience NL, Wageningen).

### Plant measurements

At each harvest, fresh root systems and rosettes were separated and weighted. Leaves were cut and counted, a leaf was defined as being larger than 1 mm [44]. Afterwards leaf area was determined using a LiCoR-3100 and/or a compact camera followed by ImageJ (Wayne Rasband, 1.51d, USA) analysis to measure leaf area. To determine dry weights, root and leaves were kept in a drying oven at 70 °C until constant weight was reached (2 to 3 days). Arabidopsis pigments, chlorophyll, and epidermal flavonol indices of expanded mature leaves were measured using FORCE-A portable fluorometer (DUALEX-SCIENTIFIC, France) and leaf stomatal conductance was measured using a leaf porometer (Decagon, SC-1, USA). The chlorophyll fluorescence measurements were carried out with a fluorcam 800 MF (Photon Systems Instruments) starting at least 2 h after dawn. For the Hoagland EC experiment (Exps. 4 and 5) on 13, 24, 31, 45 and 54 DAS photosystem II efficiency (φPSII) was measured. Actinic light in the fluorcam was set to 175 µmol m^−2^ s^−1^ (PAR), matching the PAR in the growth room. Plant acclimatized in the fluorcam measurement chamber for 3 min before measurement. On 59 DAS, plants were dark adapted for 30 min before measuring the maximum quantum yield of PSII (Fv/Fm).

### Statistical analysis

All experiments used a randomized complete block design. Blocks were distributed over two tables in a climate room; the blocks were located such that irradiance and temperature differences were minimized within the blocks. The nutrient solution composition experiment (Exp. 1) contained 3 blocks and 2 replicates (tanks) per block per harvest time (22, 28, 34, 40, 46, 50 DAS). Each replicate consisted of two plants; the first Hoagland concentration experiment (Exp. 4) contained 8 blocks with one replicate, i.e. one plant, per block per harvest time (23, 28, 33, 40, 47 DAS); the second Hoagland concentration experiment (Exp. 5) contained 7 blocks with one replicate, i.e. one plant, per block per harvest time (24, 31, 39, 45, 52, 61 DAS). All statistical analyses were done in R version 3.6.1. To test for difference between means of response variables, linear mixed effects regression models were used, i.e. the lmer function from the lme4 package version 1.1-21. For each response variable the treatment (nutrient solution) was taken as main effect with blocks as random effects. Assessment for significant differences (P > 0.05) was done using Tukey adjusted least square means (emmeans version 1.4.1). Random variables at each harvest were tested for homogeneity of variance (Levene’s test). The residuals of the lmer models were tested for normality (Shapiro–Wilkinson test and histogram inspection) and homogeneity (QQ-plots). The leaf counts data violated the assumptions required for lmer models; therefore, a Kruskal–Wallis Rank Sum Test was used, followed with a pairwise comparison to identify differences between groups. To illustrate trends in the dose response experiments smoothing-splines mixed-effects models (sme package version 1.0.2) were used. Initial smoothing parameters for the fixed-effect (lambda.mu) and random-effect function (lambda.v) for the Nelder-Mead optimisation process were chosen so that the number of knots of the spline was in approximate accordance with the data. The “AIC” was used as criteria in the optimisation process of both smoothing parameters.

The consecutive harvests from each container over time were described by the classical growth function (Eq. 1):

1 $${\text{W}}\left( t \right) = {\text{W}}_{0} e^{{{\text{t}} \cdot {\text{RGR}}}}$$

To allow for a declining relative growth rate Eq. 2 was formulated:

2a $${\text{W}}\left( t \right) = {\text{W}}_{0} e^{{{\text{t}} \cdot {\text{RGR}}_{d} }}$$

2b $${\text{RGR}}_{d} = {\text{RGR}}_{\text{slope}} \cdot t + {\text{RGR}}_{0}$$

where W(*t*) is the biomass accumulation in grams over time (*t*) in days after transfer; ${\text{W}}_{0}$ is the initial plant weight, in our case the weight at transfer; RGR is the relative growth rate; ${\text{RGR}}_{\text{slope}}$ is the slope; and ${\text{RGR}}_{0}$ the intercept, i.e. initial RGR of the declining RGR. Both Eqs. 1 and 2 were fitted using “nls” stats version 3.6.1 in R.

## Supplementary information

**Additional file 1.** Somerville and Ogren [21] solution concentration (EC) versus fresh weight [9].

**Additional file 2.** Literature search protocol.

**Additional file 3.** Dry weight accumulation over time of best performing nutrient solutions.

**Additional file 4.** Dry weight accumulation over time for all solutions.

**Additional file 5.** Pigments, chlorophyll, and epidermal flavonol indices in response to different nutrient solutions.

**Additional file 6.** Photos of plants on Murashige and Skoog (MS) solution.

**Additional file 7.** Time course of leaf area in response to NaCl dose.

**Additional file 8.** Effect of nutrient solution concentration on number of leaves.

**Additional file 9.** Example of nutrient solution pH dynamics.

**Additional file 10.** Balloon plot of nutrient solution strenght and composition.

**Additional file 11.** Photos of salt stress due to contact with nutrient solution.

**Additional file 12.** Photos of germination and seedling system.

**Additional file 13.** Chemical salts recipes used to prepare the full strenght nutrient solutions.

**Additional file 14.** R-script used to estimate the nutrient solution EC.

## Data Availability

The datasets used and/or analysed during the current study are available from the corresponding author with reasonable request.

## References

[1] Koo HL, Jenkins J, Rizzo M, Rooney T, The Arabidopsis Genome Initiative, Arabidopsis Genome Initiative S (2000). Analysis of the genome sequence of the flowering plant *Arabidopsis thaliana*. Nature.

[2] Toda T, Koyama H, Hara T (1999). A simple hydroponic culture method for the development of a highly viable root system in *Arabidopsis thaliana*. Biosci Biotechnol Biochem..

[3] Koornneef M, Meinke D (2010). The development of Arabidopsis as a model plant. Plant J..

[4] Schlesier B, Bréton F, Mock H-PP (2003). A hydroponic culture system for growing *Arabidopsis thaliana* plantlets under sterile conditions. Plant Mol Biol Rep.

[5] Smeets K, Ruytinx J, Van Belleghem F, Semane B, Lin D, Vangronsveld J (2008). Critical evaluation and statistical validation of a hydroponic culture system for *Arabidopsis thaliana*. Plant Physiol Biochem.

[6] Conn SJ, Hocking B, Dayod M, Xu B, Athman A, Henderson S (2013). Protocol: optimising hydroponic growth systems for nutritional and physiological analysis of *Arabidopsis thaliana* and other plants. Plant Methods..

[7] Huttner D, Bar-Zvi D (2003). An improved, simple, hydroponic method for growing *Arabidopsis thaliana*. Plant Mol Biol Rep.

[8] Gibeaut DM, Hulett J, Cramer GR, Seemann JR (1997). Maximal biomass of *Arabidopsis thaliana* using a simple, low-maintenance hydroponic method and favorable environmental conditions. Plant Physiol.

[9] Arteca RN, Arteca JM (2000). A novel method for growing *Arabidopsis thaliana* plants hydroponically. Physiol Plant.

[10] Siedlecka A, Krupa Z (2002). Simple method of *Arabidopsis thaliana* cultivation in liquid nutrient medium. Acta Physiol Plant.

[11] Norén H, Svensson P, Andersson B, Nore H, Svensson P, Andersson B (2004). A convenient and versatile hydroponic cultivation system for *Arabidopsis thaliana*. Physiol Plant.

[12] Alatorre-Cobos F, Calderón-Vázquez C, Ibarra-Laclette E, Yong-Villalobos L, Pérez-Torres C-A, Oropeza-Aburto A (2014). An improved, low-cost, hydroponic system for growing *Arabidopsis* and other plant species under aseptic conditions. BMC Plant Biol.

[13] Sonneveld C, Voogt W. Plant nutrition of greenhouse crops. Dordrecht: Springer Netherlands; 2009. 10.1007/978-90-481-2532-6. Accessed 20 Apr 2013.

[14] Marschner H (2011). Marschner’s mineral nutrition of higher plants.

[15] Barker AV, Pilbeam DJ. Handbook of plant nutrition, 2nd ed. In: Barker AV, Pilbeam DJ, editors. Boca Raton: CRC Press; 2015. 10.1126/science.193.4247.45.

[16] Moya C, Oyanedel E, Verdugo G, Flores MF, Urrestarazu M, Álvaro JE (2017). Increased electrical conductivity in nutrient solution management enhances dietary and organoleptic qualities in soilless culture tomato. HortScience.

[17] Gorbe E, Calatayud Á (2010). Optimization of nutrition in soilless systems: a review. Adv Bot Res.

[18] Sonneveld C, Voogt W, Spaans L, Vegetables G. A universal algorithm for calculationn of nutrient solutions. In: Proc Int Sym Grow Media Hydroponics Acta Hort 481, ISHS. 1999. p. 331–9.

[19] Dorai M, Papadopoulos A, Gosselin A, Dorais M, Papadopoulos AP. Influence of electric conductivity management on greenhouse tomato yield and fruit quality, vol. 21. 2001. p. 367–83.

[20] Tabatabaie SJ, Nazari J, Nazemiyeh H, Zehtab S, Azarmi F. Influence of various electrical conductivity levels on the growth and essential oil content of peppermint (*Menta piperita* L.) grown in hydroponic. Acta Hortic. 2007.

[21] Somerville C, Ogren W, Edelman M, Hallick RB, Chua NH (1982). Isolation of photorespiration mutants in Arabidopsis thaliana. Methods Chloroplast Biology.

[22] Hoagland DR, Arnon DI. The water-culture method for growing plants without soil. Calif Agric Exp Stn Circ. 1950.

[23] Murashige T, Skoog F (1962). A revised medium for rapid growth and bio assays with tobacco tissue cultures. Physiol Plant.

[24] Tocquin P, Corbesier L, Havelange A, Pieltain A, Kurtem E, Bernier G (2003). A novel high efficiency, low maintenance, hydroponic system for synchronous growth and flowering of *Arabidopsis thaliana*. BMC Plant Biol.

[25] Hermans C, Vuylsteke M, Coppens F, Craciun A, Inzé D, Verbruggen N (2010). Early transcriptomic changes induced by magnesium deficiency in *Arabidopsis thaliana* reveal the alteration of circadian clock gene expression in roots and the triggering of abscisic acid-responsive genes. New Phytol.

[26] Marschner P (2012). Marschner’s mineral nutrition of higher plants.

[27] Nathoo N, MacDonald J, Weselowski B, Yuan ZC (2019). Comparative transcriptomic analysis reveals different responses of *Arabidopsis thaliana* roots and shoots to infection by *Agrobacterium tumefaciens* in a hydroponic co-cultivation system. Physiol Mol Plant Pathol.

[28] Kowalchuk GA, Stephen JR (2001). Ammonia-oxidizing bacteria: a model for molecular microbial ecology. Annu Rev Microbiol.

[29] Munns R, Tester M (2008). Mechanisms of salinity tolerance. Annu Rev Plant Biol.

[30] Cramer GR (2002). Response of abscisic acid mutants of Arabidopsis to salinity. Funct Plant Biol.

[31] Raven JA (2016). Chloride: essential micronutrient and multifunctional beneficial ion. J Exp Bot.

[32] Pilon-Smits EA, Quinn CF, Tapken W, Malagoli M, Schiavon M (2009). Physiological functions of beneficial elements. Curr Opin Plant Biol.

[33] Nazarideljou MJ, van Delden S, Marcelis L (2019). Optimization of *Arabidopsis* germination system for nutritional studies under soilless culture. Hortic Plant Nutr..

[34] Karssemeijer PN, Reichelt M, Gershenzon J, van Loon J, Dicke M (2020). Foliar herbivory by caterpillars and aphids differentially affects phytohormonal signalling in roots and plant defence to a root herbivore. Plant Cell Environ..

[35] Shi WM, Muramoto Y, Ueda A, Takabe T (2001). Cloning of peroxisomal ascorbate peroxidase gene from barley and enhanced thermotolerance by overexpressing in *Arabidopsis thaliana*. Gene.

[36] Matsuyama T, Tamaoki M, Nakajima N, Aono M, Kubo A, Moriya S (2002). cDNA microarray assessment for ozone-stressed *Arabidopsis thaliana*. Environ Pollut.

[37] Iwabuchi A, Katte N, Suwa M, Goto J, Inui H (2020). Factors regulating the differential uptake of persistent organic pollutants in cucurbits and non-cucurbits. J Plant Physiol.

[38] Egusa M, Matsukawa S, Miura C, Nakatani S, Yamada J, Endo T (2020). Improving nitrogen uptake efficiency by chitin nanofiber promotes growth in tomato. Int J Biol Macromol..

[39] Schwarz D, Gross W (2004). Algae affecting lettuce growth in hydroponic systems. J Hortic Sci Biotechnol..

[40] Lange B, van der Ent A, Baker AJM, Echevarria G, Mahy G, Malaisse F (2017). Copper and cobalt accumulation in plants: a critical assessment of the current state of knowledge. New Phytol.

[41] Dilworth MJ, Robson AD, Chatel DL (1979). Cobalt and nitrogen fixation in *Lupinus angustifolius* L. II. nodule formation and function. New Phytol.

[42] Kliewer M, Evans HJ (1963). Identification of cobamide coenzyme in nodules of symbionts & isolation of the B 12 coenzyme from *Rhizobium meliloti*. Plant Physiol.

[43] Kliewer M, Evans HJ (1963). Cobamide coenzyme contents of soybean nodules & nitrogen fixing bacteria in relation to physiological conditions. Plant Physiol.

[44] Boyes DC, Zayed AM, Ascenzi R, McCaskill AJ, Hoffman NE, Davis KR (2001). Growth stage-based phenotypic analysis of *Arabidopsis*: a model for high throughput functional genomics in plants. Plant Cell.

