# Supplementary material for: Nutrient solutions for Arabidopsis thaliana: a study on nutrient solution composition in hydroponics systems
Source: Plant Methods. 2020 May 18;16:72. doi: 10.1186/s13007-020-00606-4 (PMC7324969; doi:10.1186/s13007-020-00606-4)
Supplement: Supplementary file 1 — Additional file 1. Somerville and Ogren [21] solution concentration (EC) versus fresh weight [9]. [file 13007_2020_606_MOESM1_ESM.docx]

Additional file 1: Somerville and Ogren (1982) solution from Arteca and Arteca (2000)


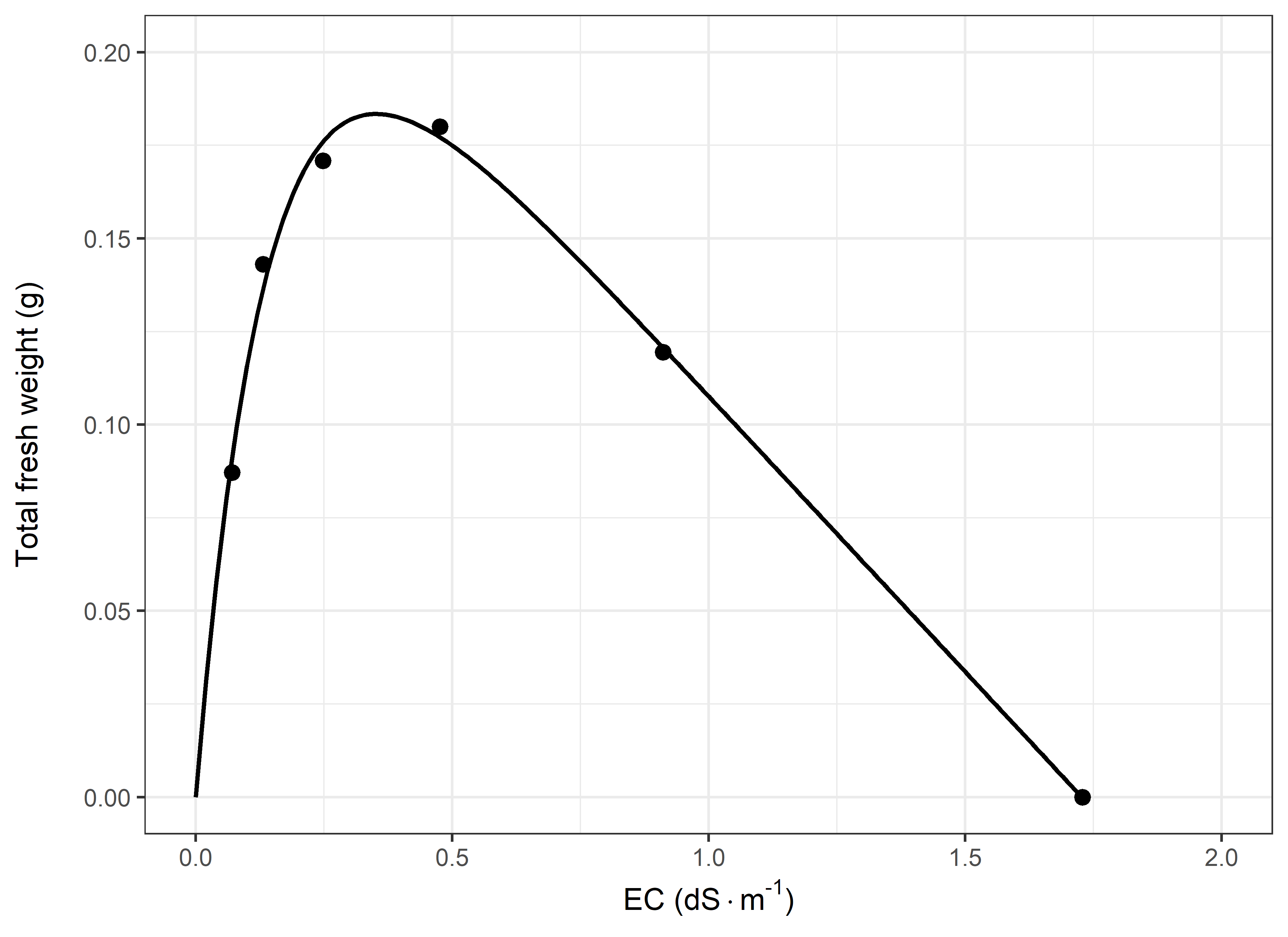


Fig. S1. Redrawing of the bar graphs that showed fresh weight in response to Somerville nutrient solution concentration (Arteca and Arteca [9]) to a dose response curve of nutrient solution concentration expressed in EC (dS m^-1^ ) versus fresh weight (g). Fitted with an optimum curve $\boldsymbol{W(EC)=a\cdot}\left( \boldsymbol{1-}\boldsymbol{e}^{\boldsymbol{-}\mathbf{b}\boldsymbol{\cdot EC}} \right)\boldsymbol{\cdot}\left( \boldsymbol{-EC+}\mathbf{EC}_{\mathbf{max}} \right)$ were *W* is plant weight in grams, $\boldsymbol{a}$ and $\boldsymbol{b}$ determine the slope and optimum of the curve and $\mathbf{EC}_{\mathbf{max}}$is the EC at which the solution is lethal.
