# Supplementary material for: Nutrient solutions for Arabidopsis thaliana: a study on nutrient solution composition in hydroponics systems
Source: Plant Methods. 2020 May 18;16:72. doi: 10.1186/s13007-020-00606-4 (PMC7324969; doi:10.1186/s13007-020-00606-4)
Supplement: Supplementary file 2 — Additional file 2. Literature search protocol. [file 13007_2020_606_MOESM2_ESM.docx]

Additional file 2: Literature search protocol

We used the Textpresso tool to do a systematic literature scan searching the full text of the material and methods of 21070 Arabidopsis articles (http://www.textpresso.org/Arabidopsis/). This returned 811 articles, screening these articles revealed that 620 actually cultivated Arabidopsis hydroponically. Based on this survey we chose the most frequently used solutions: Hoagland and Arnon [22], Murashige and Skoog [23], Tocquin [24], Hermans [25], and Conn [6].

This survey was done in 2015, before we started our experiments on nutrient solutions for Arabidopsis. Since 2015 the textpresso database has not been updated. To check if our choice of solutions is still actual we did a second search in scopus with the following search criteria: ( TITLE-ABS-KEY ( Arabidopsis )  AND  TITLE-ABS-KEY ( hydroponic*  OR  "water culture*" ) )  AND  ( LIMIT-TO ( PUBYEAR ,  2018 )  OR  LIMIT-TO ( PUBYEAR ,  2017 )  OR  LIMIT-TO ( PUBYEAR ,  2016 )  OR  LIMIT-TO ( PUBYEAR ,  2015 ) )

This returned 120 papers of which 90 were actually using a hydroponic system. From these 90 studies 30 were using Hoagland or Hoagland based solutions. A surprisingly high number of 23 studies was using Murashige and Skoog (MS) or MS based solution.
