# Supplementary material for: Nutrient solutions for Arabidopsis thaliana: a study on nutrient solution composition in hydroponics systems
Source: Plant Methods. 2020 May 18;16:72. doi: 10.1186/s13007-020-00606-4 (PMC7324969; doi:10.1186/s13007-020-00606-4)
Supplement: Supplementary file 3 — Additional file 3. Dry weight accumulation over time of best performing nutrient solutions. [file 13007_2020_606_MOESM3_ESM.docx]

Additional file 3: Dry weight accumulation over time of best performing nutrient solutions.


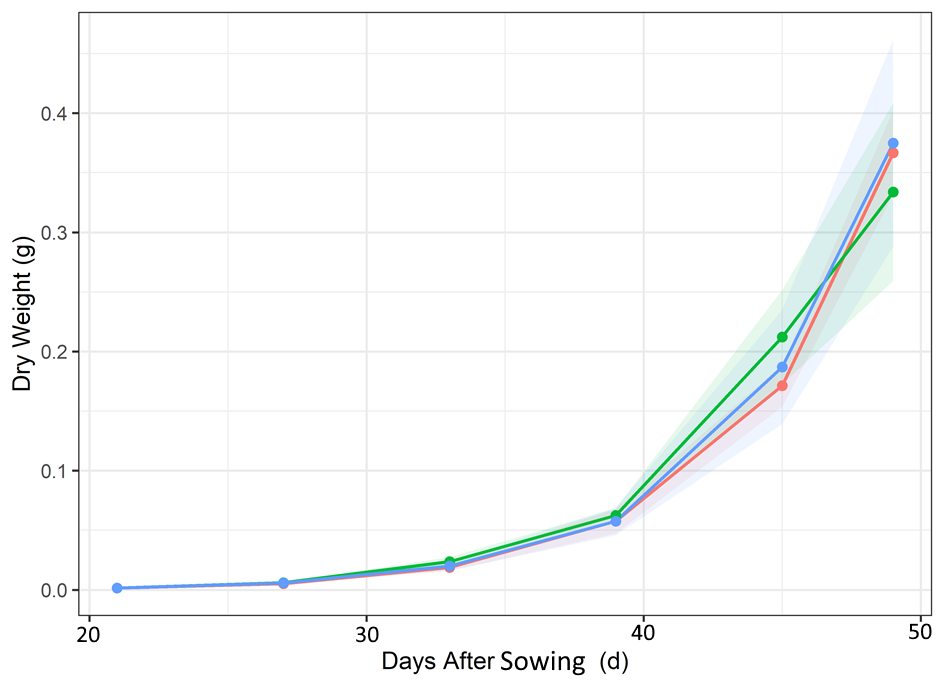


Fig. S3. Dry weight accumulation over time of the three best performing nutrient solutions (green dots: Hoagland and Arnon [22], blue dots: Conn *et al*., [6] and red dots: Tocquin *et al*., [24]. Lines with the same colour interpolate the data between the data points (means, n = 6). Transparent bands indicate the interpolated confidence interval of the data points with the same colour.
