# Supplementary material for: Nutrient solutions for Arabidopsis thaliana: a study on nutrient solution composition in hydroponics systems
Source: Plant Methods. 2020 May 18;16:72. doi: 10.1186/s13007-020-00606-4 (PMC7324969; doi:10.1186/s13007-020-00606-4)

Additional file 4: Dry weight accumulation over time for all solutions


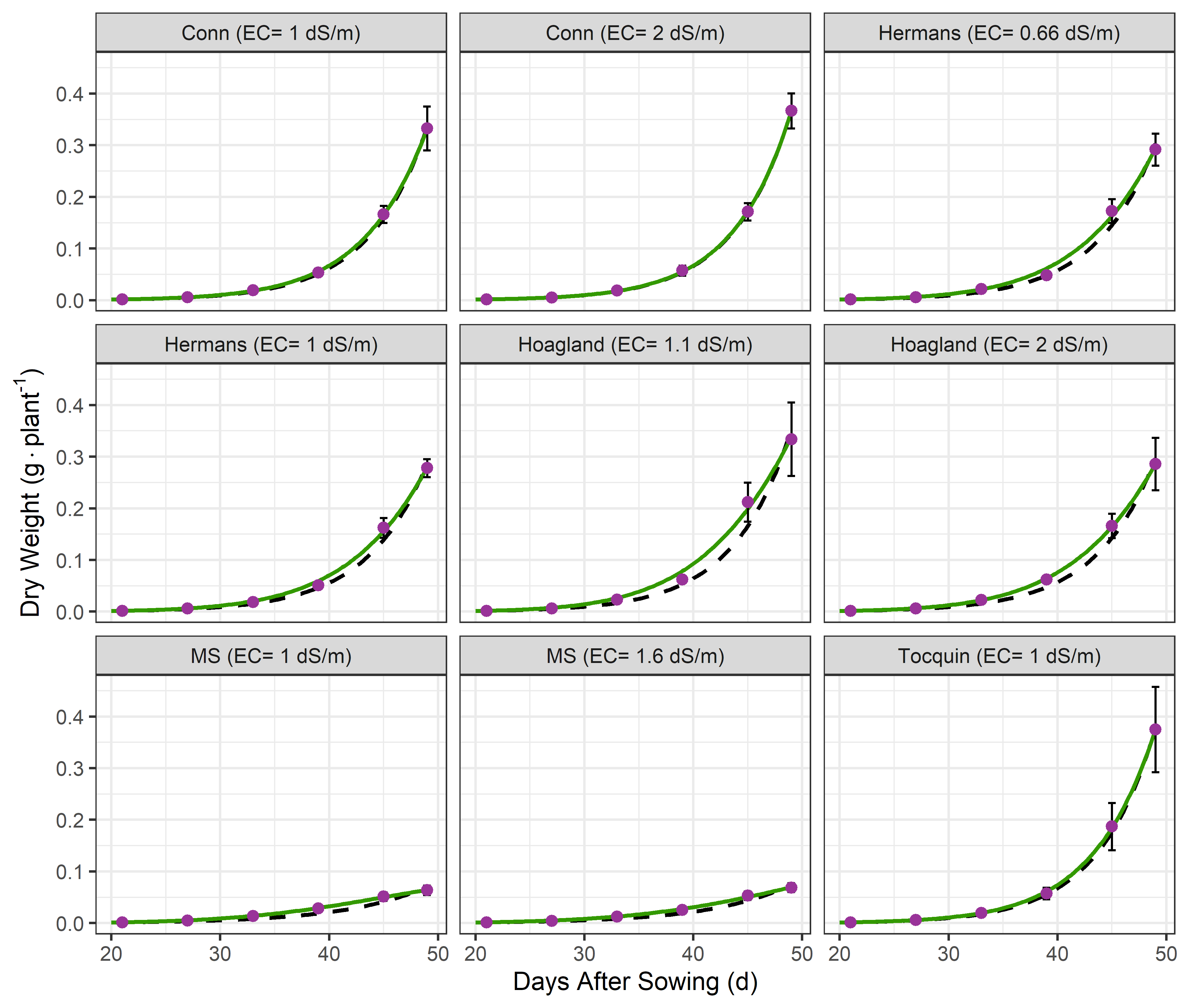


Fig. S4-A. The mean relative growth rate (RGR) of Arabidopsis rosettes (circles) grown in different nutrient solutions (n = 6) (EXP. 1). Error bar represents the standard deviation of the mean. Constant growth rate (black dashed line, eq. 1) and linearly declining growth rate (solid green line, eq. 2) fitted on dry weight data of all replicates per treatment.


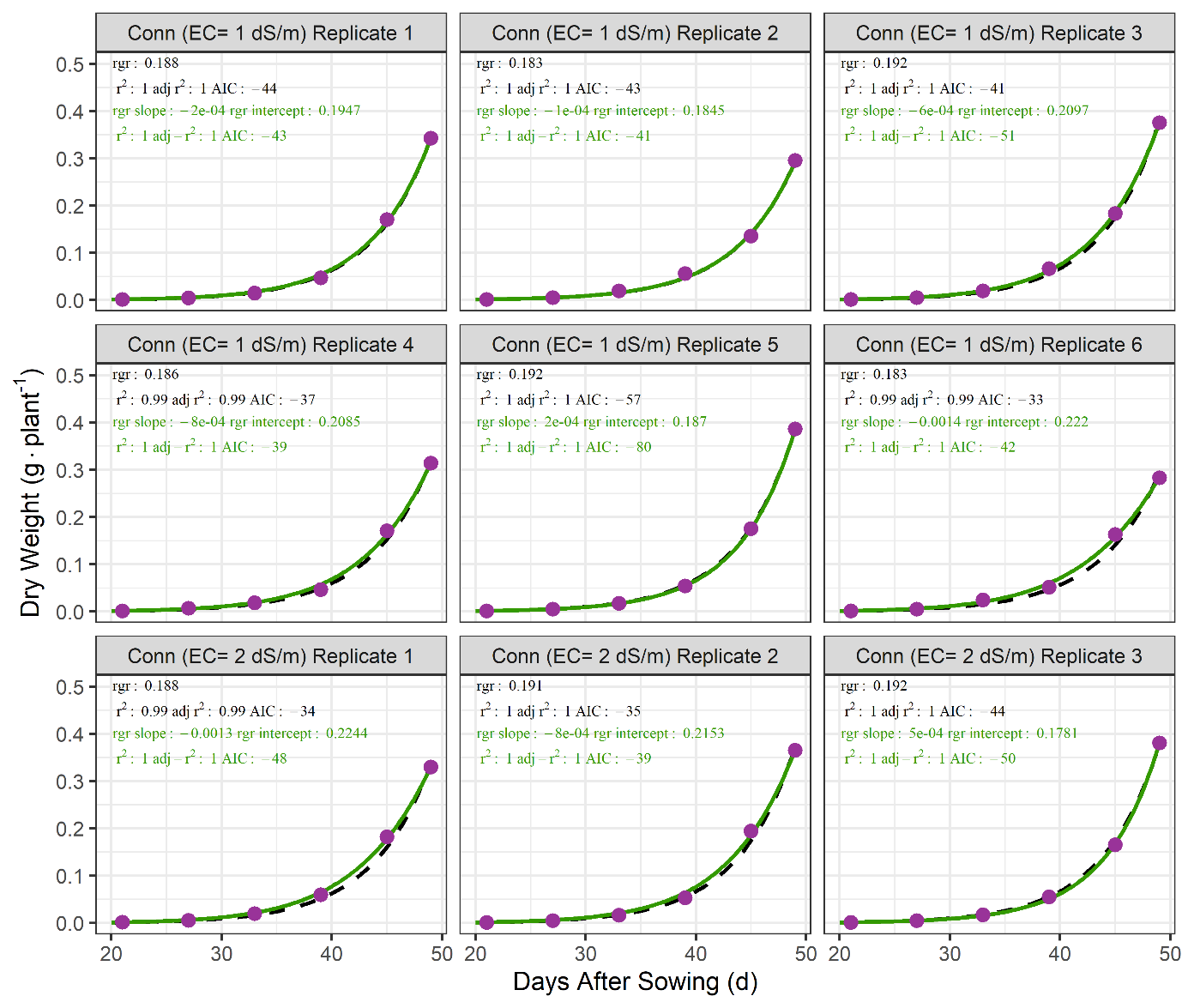


Fig. S4-B1 of 6. Constant growth rate (black dashed line, eq. 1) and linearly declining growth rate (solid green line, eq. 2) fitted on dry weight data per replicate per treatment.


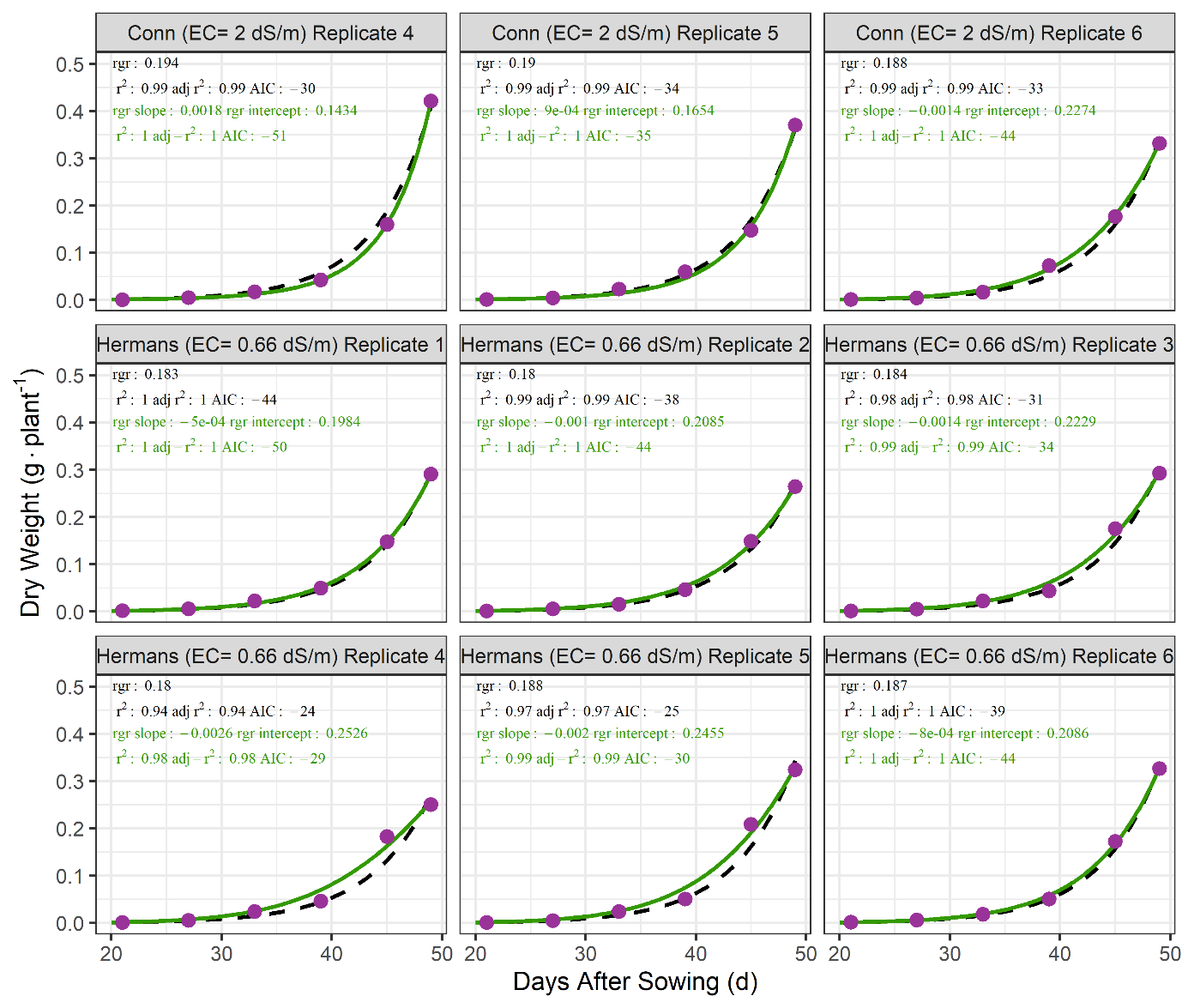


Fig. S4-B2 of 6. Constant growth rate (black dashed line, eq. 1) and linearly declining growth rate (solid green line, eq. 2) fitted on dry weight data per replicate per treatment.


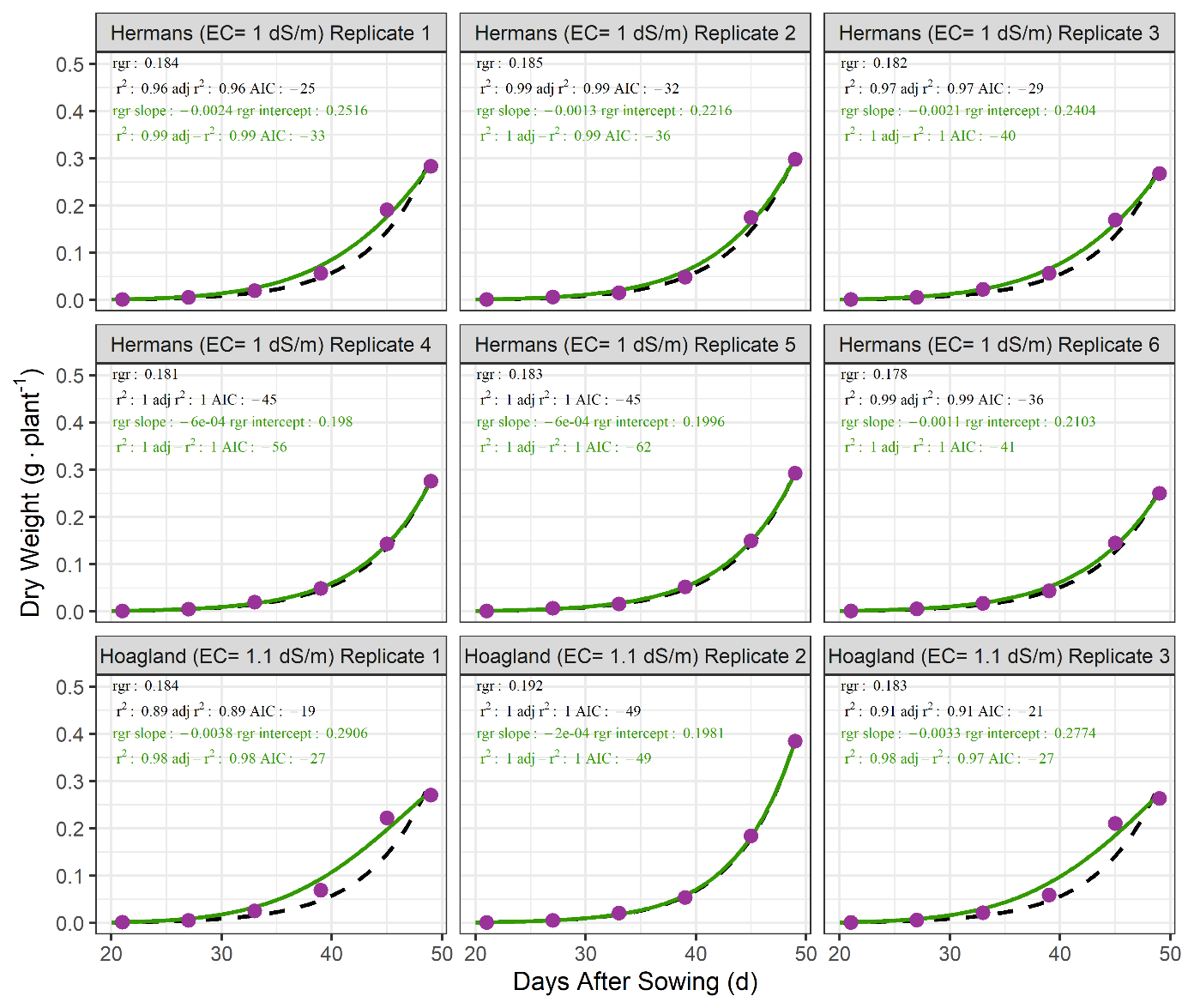


Fig. S4-B3 of 6. Constant growth rate (black dashed line, eq. 1) and linearly declining growth rate (solid green line, eq. 2) fitted on dry weight data per replicate per treatment.


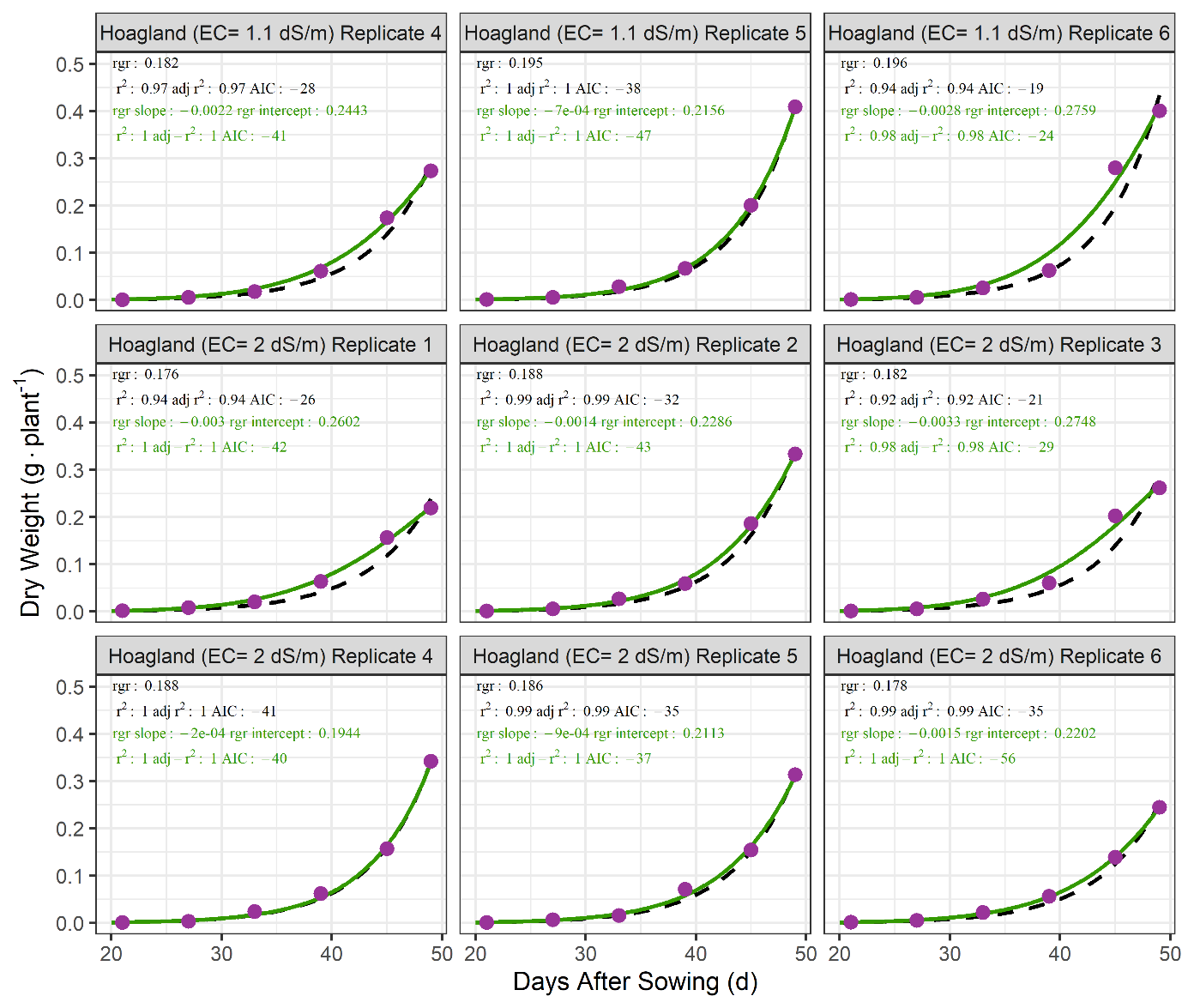


Fig. S4-B4 of 6. Constant growth rate (black dashed line, eq. 1) and linearly declining growth rate (solid green line, eq. 2) fitted on dry weight data per replicate per treatment.


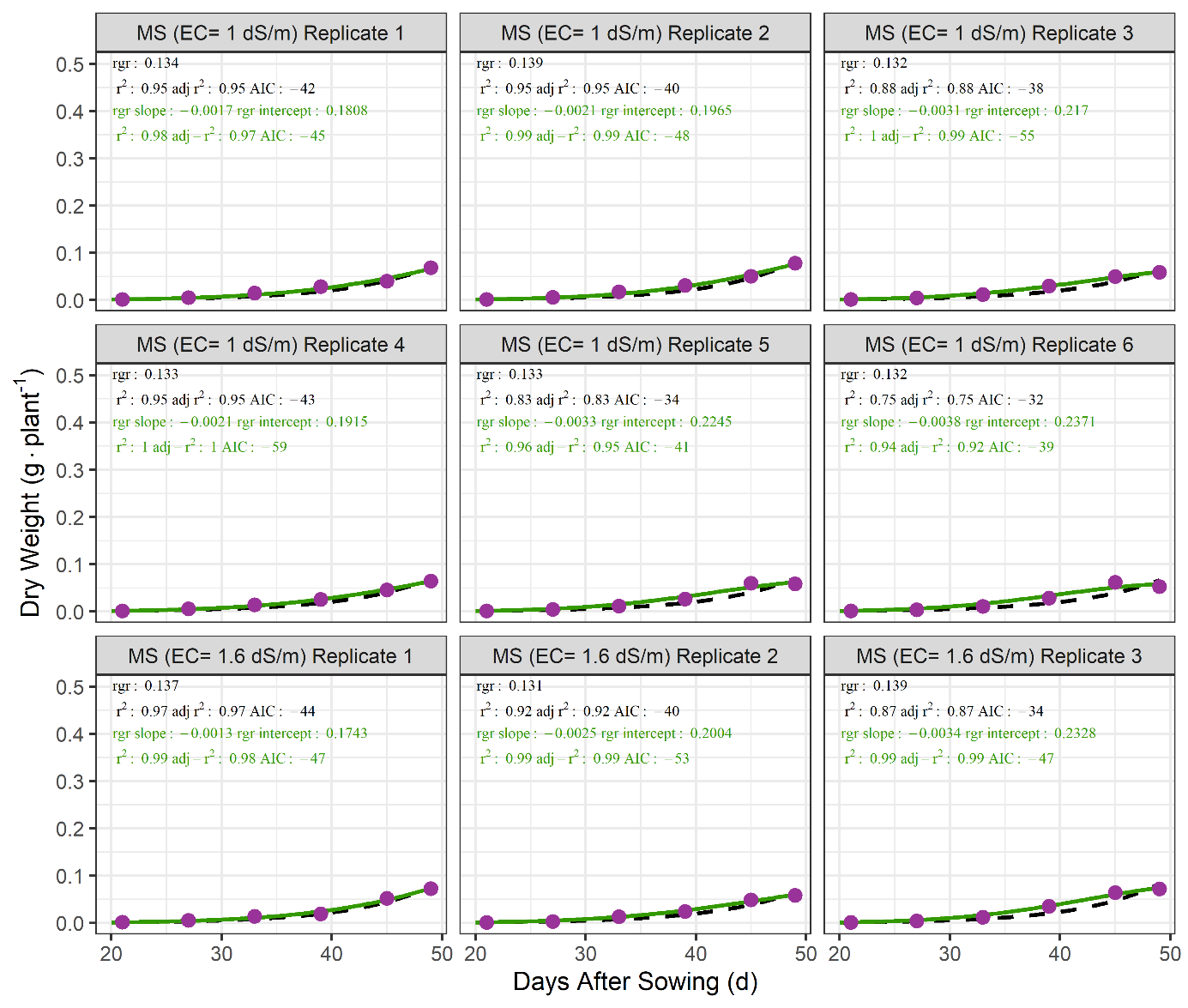


Fig. S4-B5 of 6. Constant growth rate (black dashed line, eq. 1) and linearly declining growth rate (solid green line, eq. 2) fitted on dry weight data per replicate per treatment.


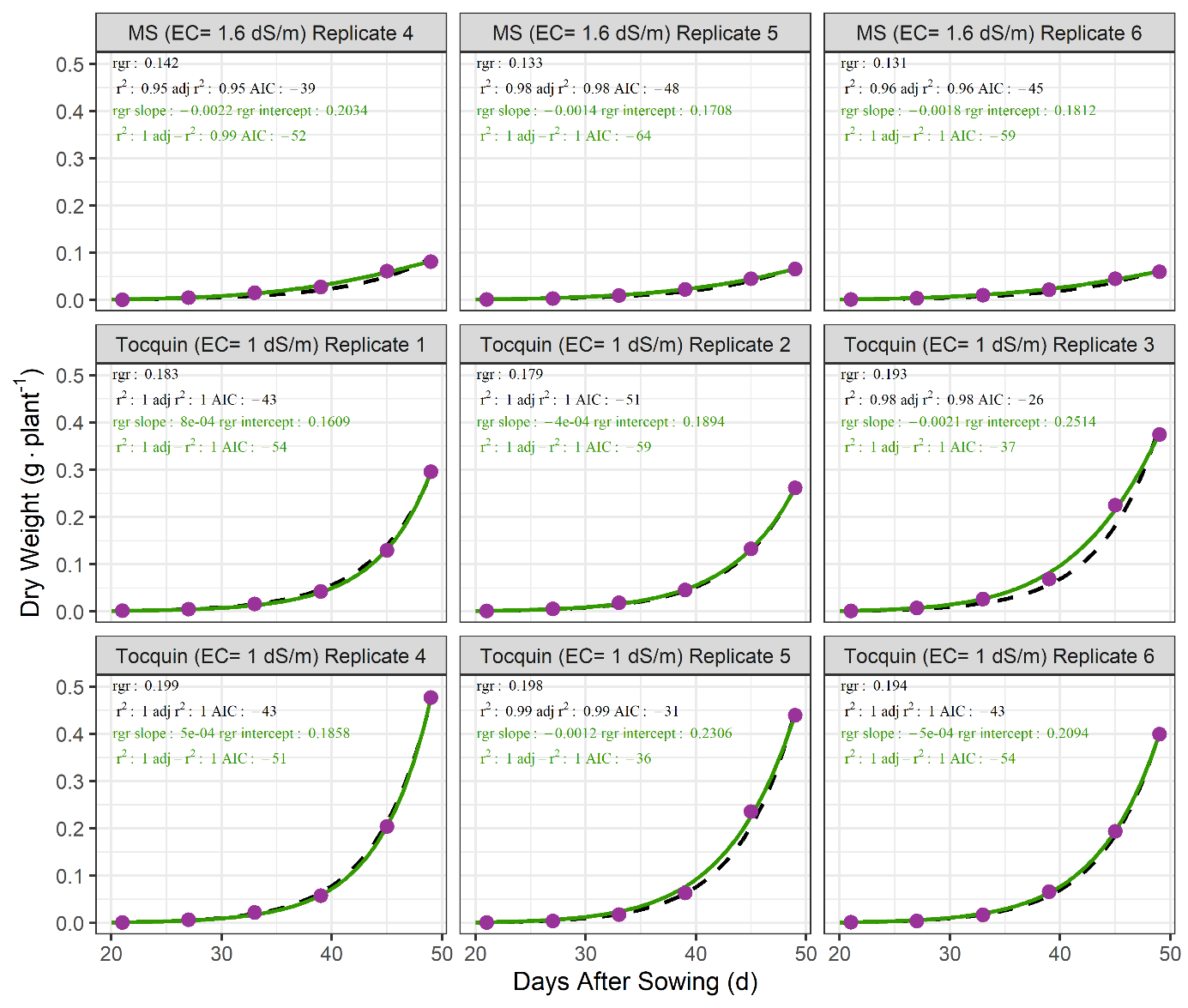


Fig. S4-B6 of 6. Constant growth rate (black dashed line, eq. 1) and linearly declining growth rate (solid green line, eq. 2) fitted on dry weight data per replicate per treatment.


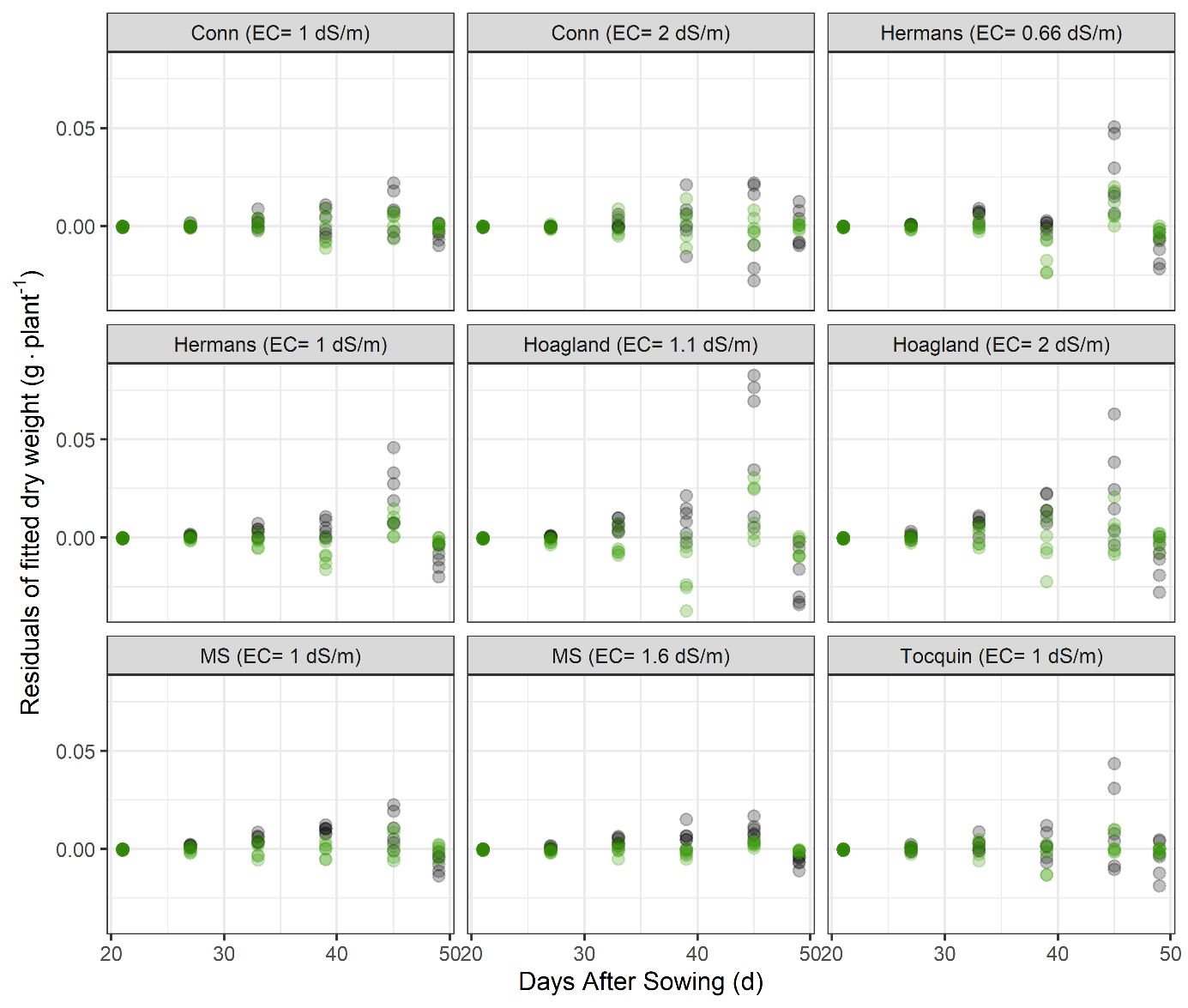


Fig. S4-C. Residuals of dry weight data modelled using a constant growth rate (grey dots, eq. 1) and linearly declining growth rate (green dots, eq. 2).

Parameter $W_{0}$ in eq. 2 is theoretically equal for all plants in the experiment and was therefore set to its mean value of 0.00146 g measured at transfer for easier comparisson. Parameter $\mathrm{RGR}_{0}$ in eq. 2 is the intercept of the slope of decline in RGR, i.e. parameter $\mathrm{RGR}_{\mathrm{slope}}$. With a mean value of 0.214 g plant^-1^ d^-1^ d^-1^ parameter $\mathrm{RGR}_{0}$ was higher than the average RGR of 0.175 g plant^-1^ d^-1^ d^-1^ fitted with the traditional model (Table S4).


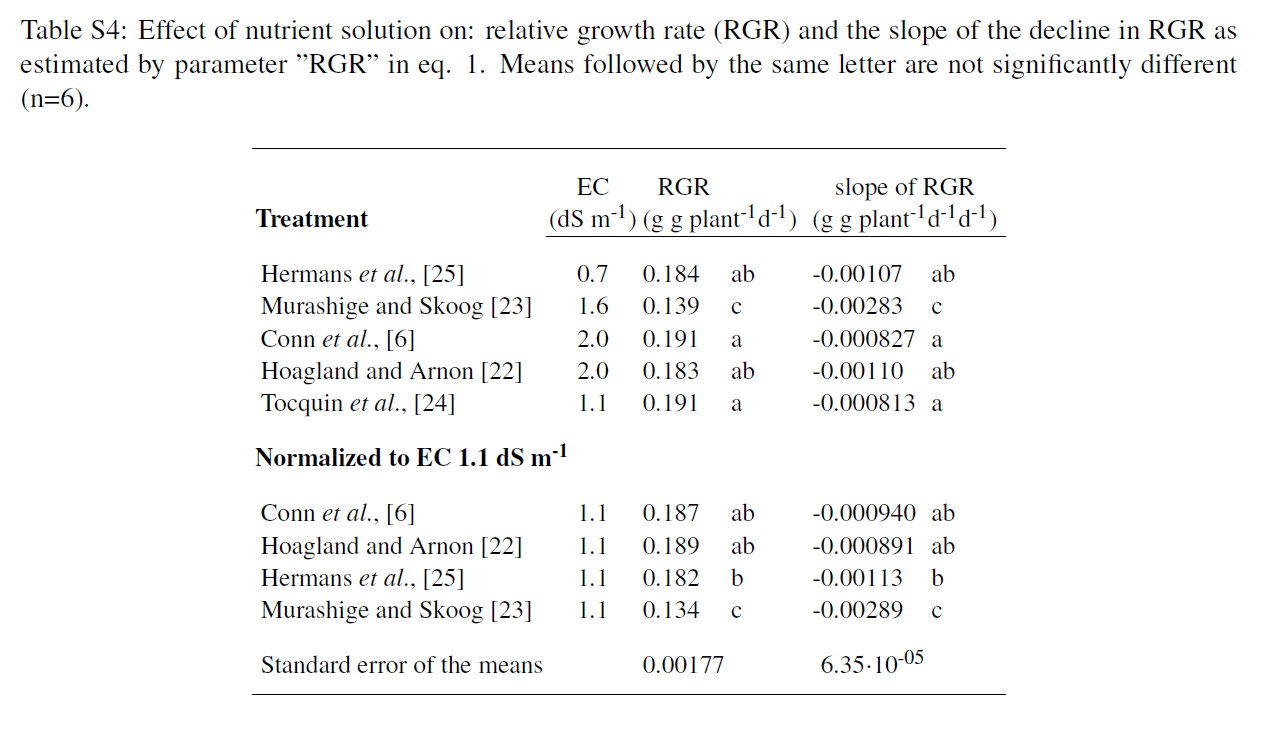

Supplement: Supplementary file 4 — Additional file 4. Dry weight accumulation over time for all solutions. [file 13007_2020_606_MOESM4_ESM.docx]
