# Supplementary material for: Nutrient solutions for Arabidopsis thaliana: a study on nutrient solution composition in hydroponics systems
Source: Plant Methods. 2020 May 18;16:72. doi: 10.1186/s13007-020-00606-4 (PMC7324969; doi:10.1186/s13007-020-00606-4)
Supplement: Supplementary file 5 — Additional file 5. Pigments, chlorophyll, and epidermal flavonol indices in response to different nutrient solutions. [file 13007_2020_606_MOESM5_ESM.docx]

Additional file 5: Dualex® measurements


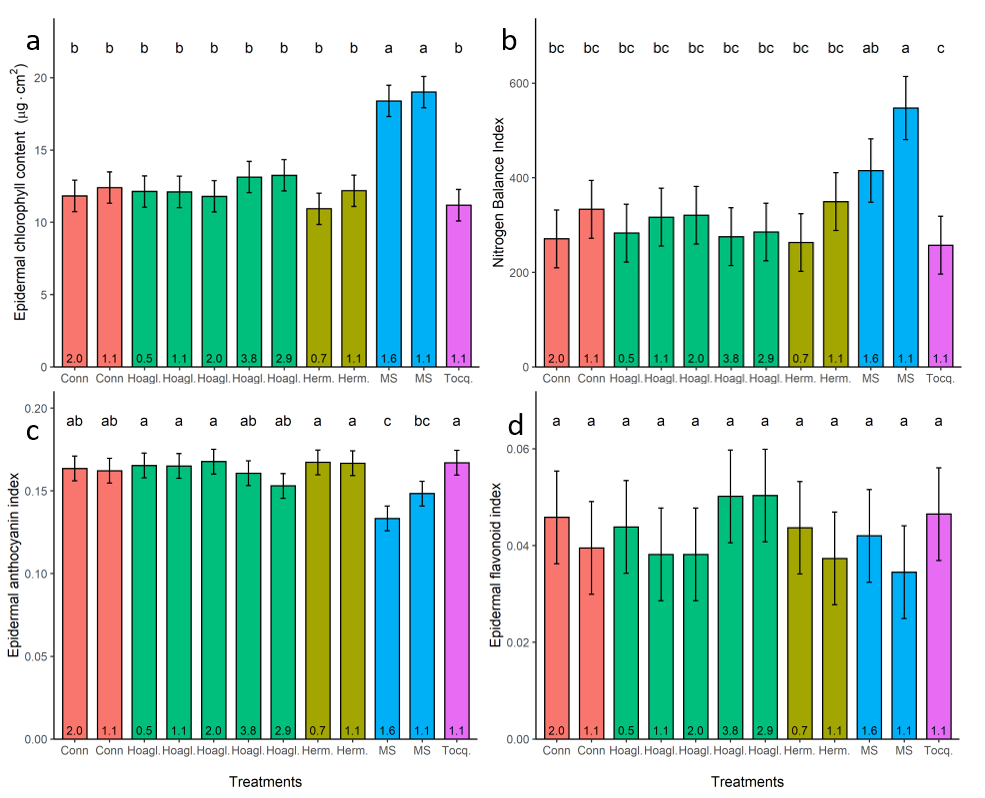


Fig. S5: Length of the bars correspond to the means (n=6) of: (a) chlorophyll index (b) Nitrogen balance index (c) anthocyanin index and (d) flavonoid index as measure with the Dualex®. Different colours are identifiers of different nutrient solutions. Error bar represent the 95% confidence intervals of the means. Numbers at the bottom of the bars indicate nutrient solution concentration (EC value in dS m^-1^).
