# Supplementary material for: Nutrient solutions for Arabidopsis thaliana: a study on nutrient solution composition in hydroponics systems
Source: Plant Methods. 2020 May 18;16:72. doi: 10.1186/s13007-020-00606-4 (PMC7324969; doi:10.1186/s13007-020-00606-4)
Supplement: Supplementary file 6 — Additional file 6. Photos of plants on Murashige and Skoog (MS) solution. [file 13007_2020_606_MOESM6_ESM.docx]

Additional file 6: Plants on Murashige and Skoog (MS) solution


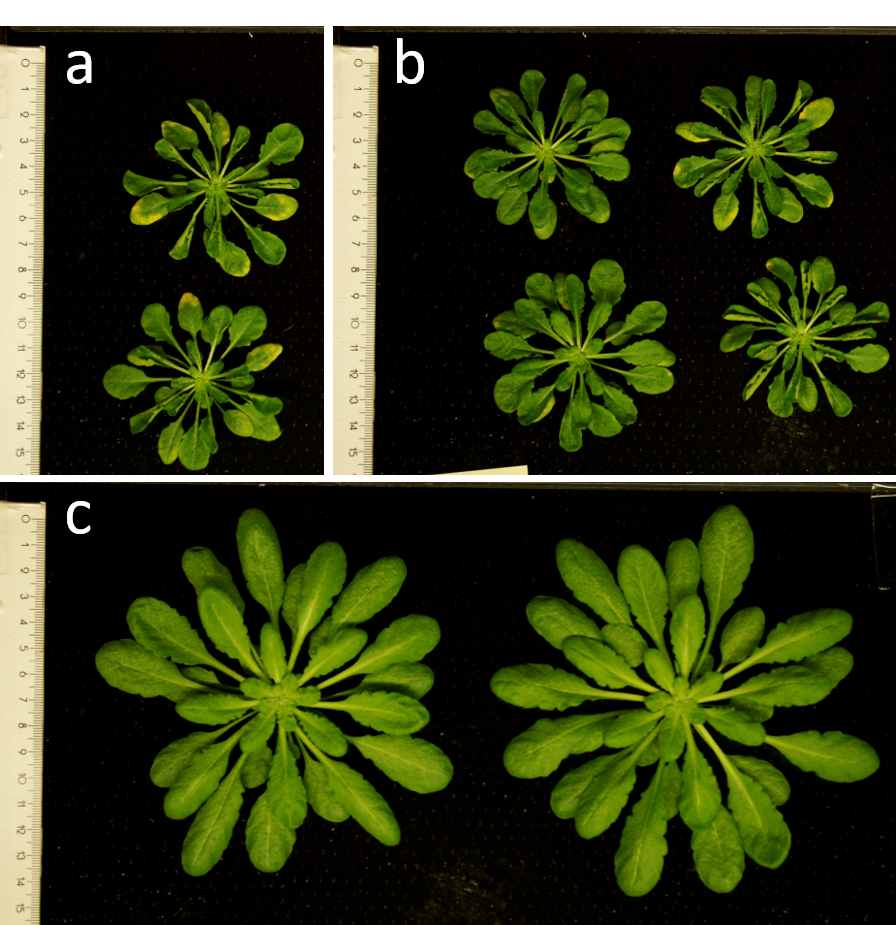


Fig S6. Arabidopsis plants 45 DAS grown on (a) Murashige and Skoog [23] 1.1 dS m^-1^ , (b) ¼ x Murashige and Skoog [23] 1.6 dS m^-1^ and (c) full concentration Tocquin *et al*., [24] solution 1.1 dS m^-1^.
