# Supplementary material for: Nutrient solutions for Arabidopsis thaliana: a study on nutrient solution composition in hydroponics systems
Source: Plant Methods. 2020 May 18;16:72. doi: 10.1186/s13007-020-00606-4 (PMC7324969; doi:10.1186/s13007-020-00606-4)
Supplement: Supplementary file 7 — Additional file 7. Time course of leaf area in response to NaCl dose. [file 13007_2020_606_MOESM7_ESM.docx]

Additional file 7: Time course of leaf area in response to NaCl dose


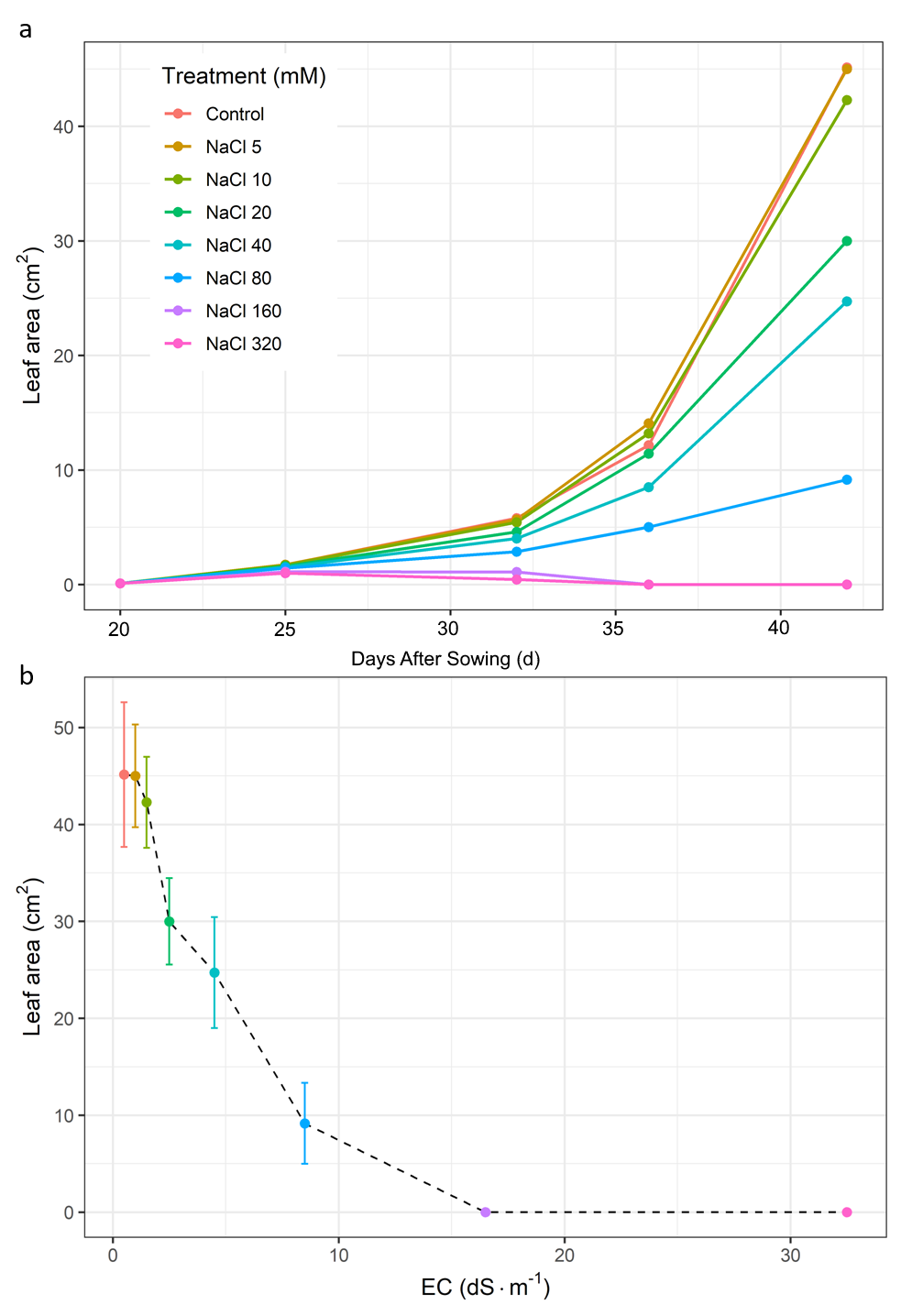


Fig. S7. (a) Leaf area (cm^2^) as a function of time and (b) as a function of EC (22 DAT) for addition of 0,5,10,20,40,80,160 and 320 mM NaCl to 0.5 dS m^-1^ EC Hoagland solution.
