# Supplementary material for: Nutrient solutions for Arabidopsis thaliana: a study on nutrient solution composition in hydroponics systems
Source: Plant Methods. 2020 May 18;16:72. doi: 10.1186/s13007-020-00606-4 (PMC7324969; doi:10.1186/s13007-020-00606-4)
Supplement: Supplementary file 8 — Additional file 8. Effect of nutrient solution concentration on number of leaves. [file 13007_2020_606_MOESM8_ESM.docx]

Additional file 8: Effect of nutrient solution concentration on number of leaves


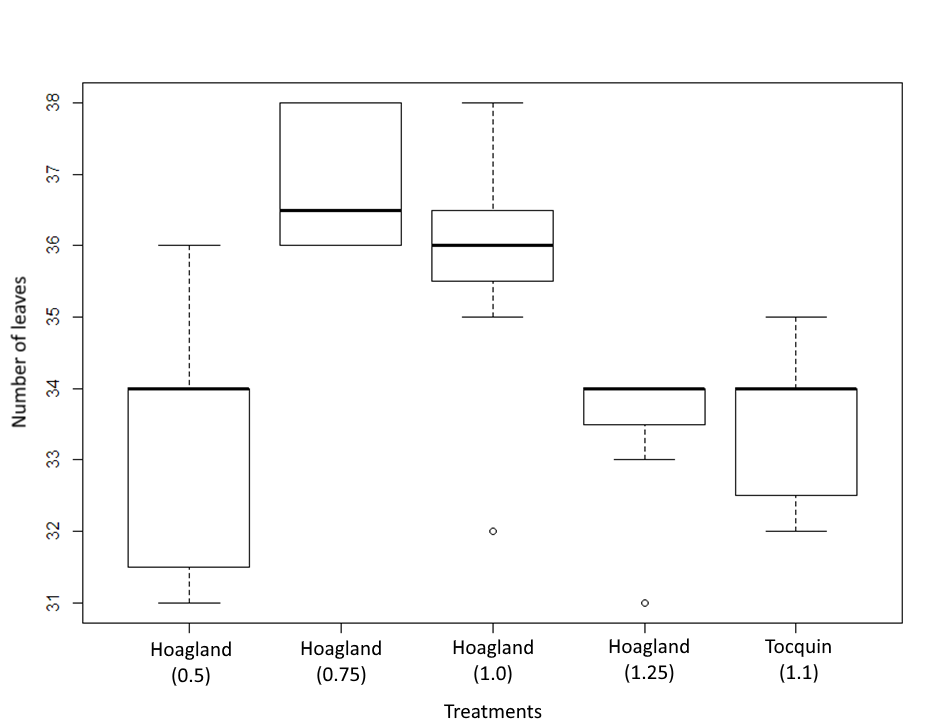


Fig S8. A boxplot of leaf numbers from the high-resolution Hoagland EC response experiment. The nutrient solution name is followed by the EC in dS m^-1^ in brackets. Data of leaf counts violated the assumptions required for lmer models, therefore a Kruskal-Wallis Rank Sum Test was used followed by a pairwise comparison which identified that the Hoagland EC 0.75 and Hoagland EC 1 have a higher leaf number than the other treatments.
