# Supplementary material for: Nutrient solutions for Arabidopsis thaliana: a study on nutrient solution composition in hydroponics systems
Source: Plant Methods. 2020 May 18;16:72. doi: 10.1186/s13007-020-00606-4 (PMC7324969; doi:10.1186/s13007-020-00606-4)
Supplement: Supplementary file 9 — Additional file 9. Example of nutrient solution pH dynamics. [file 13007_2020_606_MOESM9_ESM.docx]

Additional file 9: Example of nutrient solution pH dynamics


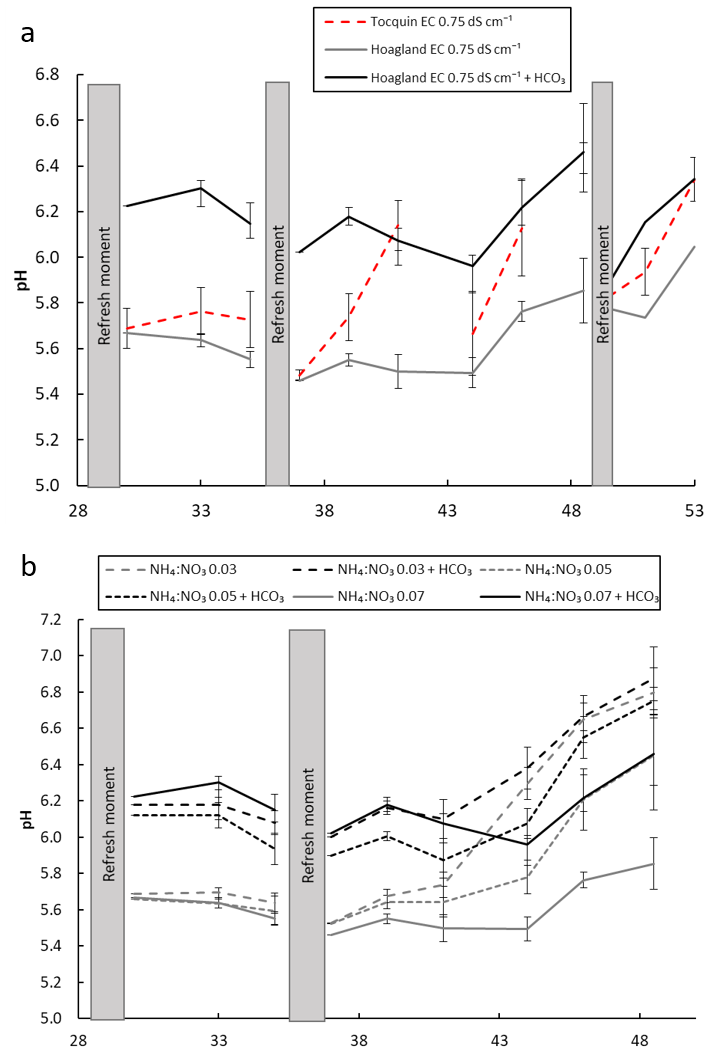


**Time (d)**

Fig. S9. (a) pH change over time for Tocquin, Hoagland solution and Hoagland solution with 0.5 mmol L^-1^ HCO_3_. (b) pH change over time for different NH_4_^+^ : NO_3_^-^ ratio’s in Hoagland solution with and without HCO_3_.
