# Supplementary material for: Nutrient solutions for Arabidopsis thaliana: a study on nutrient solution composition in hydroponics systems
Source: Plant Methods. 2020 May 18;16:72. doi: 10.1186/s13007-020-00606-4 (PMC7324969; doi:10.1186/s13007-020-00606-4)
Supplement: Supplementary file 10 — Additional file 10. Balloon plot of nutrient solution strenght and composition. [file 13007_2020_606_MOESM10_ESM.docx]

Additional file 10: Balloon plot nutrient solutions


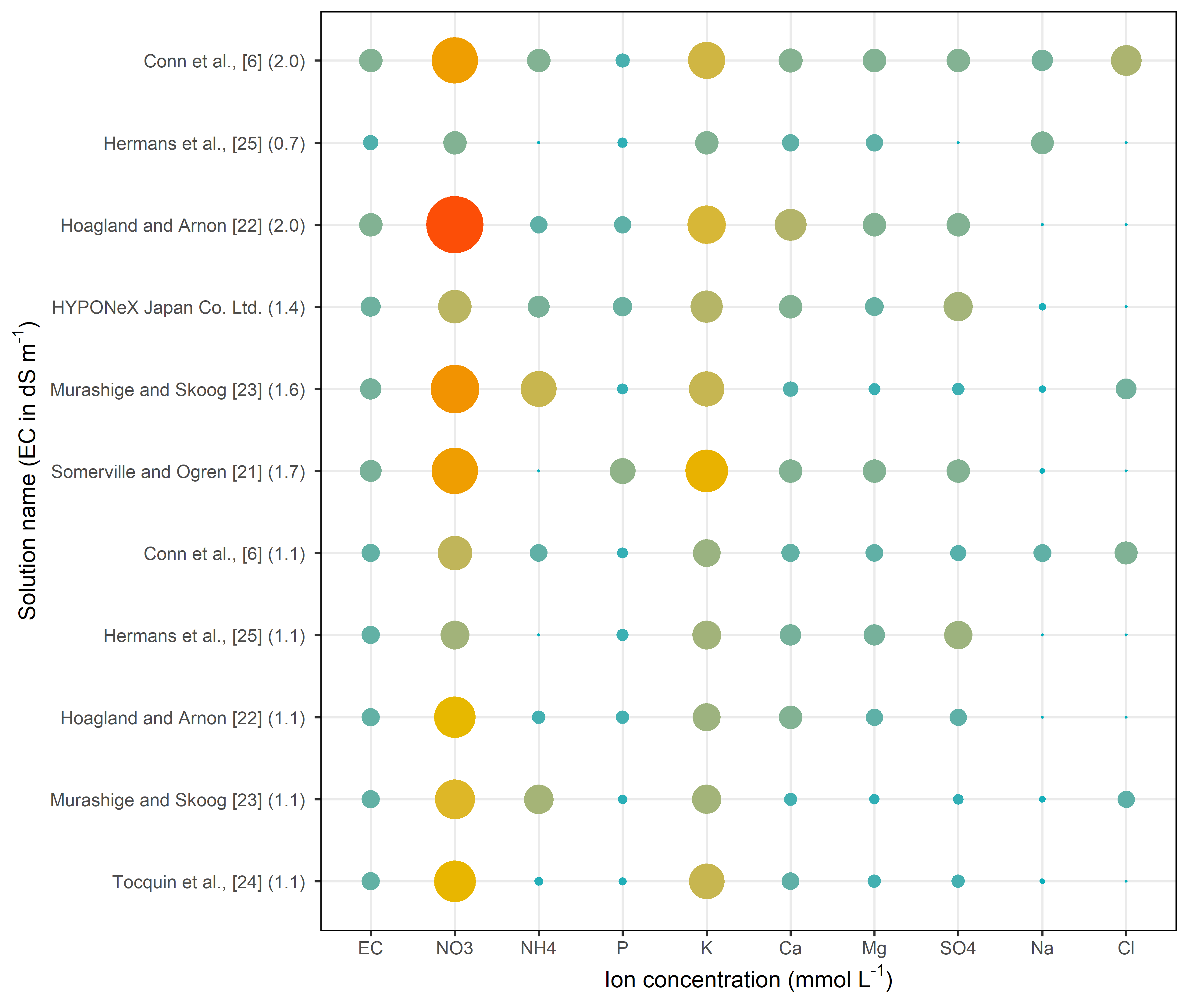


Fig. S10. Balloon plot of nutrient solution composition for intuitive visible comparison, nutrient solution names are followed by their (EC) in dS m^-1^. Balloon sizes are normalized on the maximal value (14).
