# Supplementary material for: Nutrient solutions for Arabidopsis thaliana: a study on nutrient solution composition in hydroponics systems
Source: Plant Methods. 2020 May 18;16:72. doi: 10.1186/s13007-020-00606-4 (PMC7324969; doi:10.1186/s13007-020-00606-4)
Supplement: Supplementary file 11 — Additional file 11. Photos of salt stress due to contact with nutrient solution. [file 13007_2020_606_MOESM11_ESM.docx]

Additional file 11: Salt stress due to contact with nutrient solution


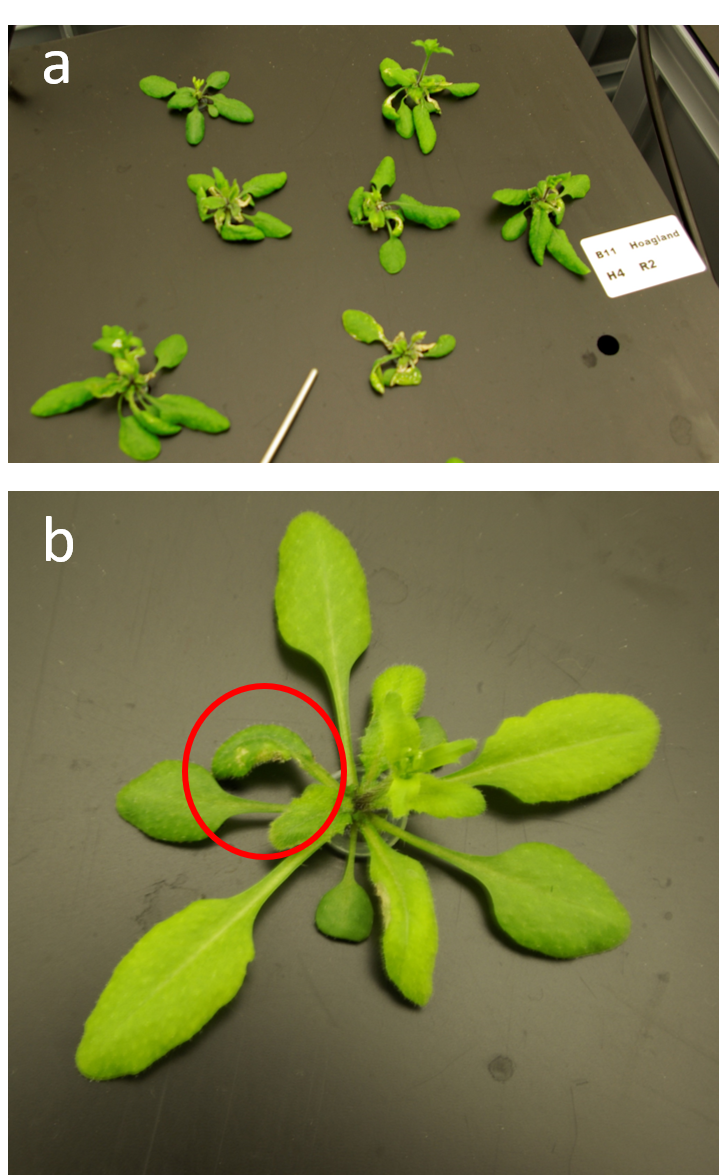


Fig S11. Damaged Arabidopsis plants with their microtubes in contact with the nutrient solution. (a) Overview off a tank with full Hoagland solution (EC = 2 dS m^-1^), (b) close up with curled leave due salt stress. For second opinion and to check if symptoms were not caused by any kind of pathogen, plants were examined by the plant “doctor facility” of Eurofins Agroscience NL, Wageningen (research ref: 724620/003797564). After microscopic investigation and incubation on growth media no fungi pathogenic spores, pathogenic bacteria nor mites were found. The high concentration is a result of rapid evaporation of the nutrient solution that was “flowing” trough the agar in the microtube tube. In subsequent trials where after transfer (20 DAS) the agar was not in contact with the nutrient solution these symptoms were no longer observed.
