# Supplementary material for: Nutrient solutions for Arabidopsis thaliana: a study on nutrient solution composition in hydroponics systems
Source: Plant Methods. 2020 May 18;16:72. doi: 10.1186/s13007-020-00606-4 (PMC7324969; doi:10.1186/s13007-020-00606-4)
Supplement: Supplementary file 12 — Additional file 12. Photos of germination and seedling system. [file 13007_2020_606_MOESM12_ESM.docx]

Additional file 12: Germination and seedling system


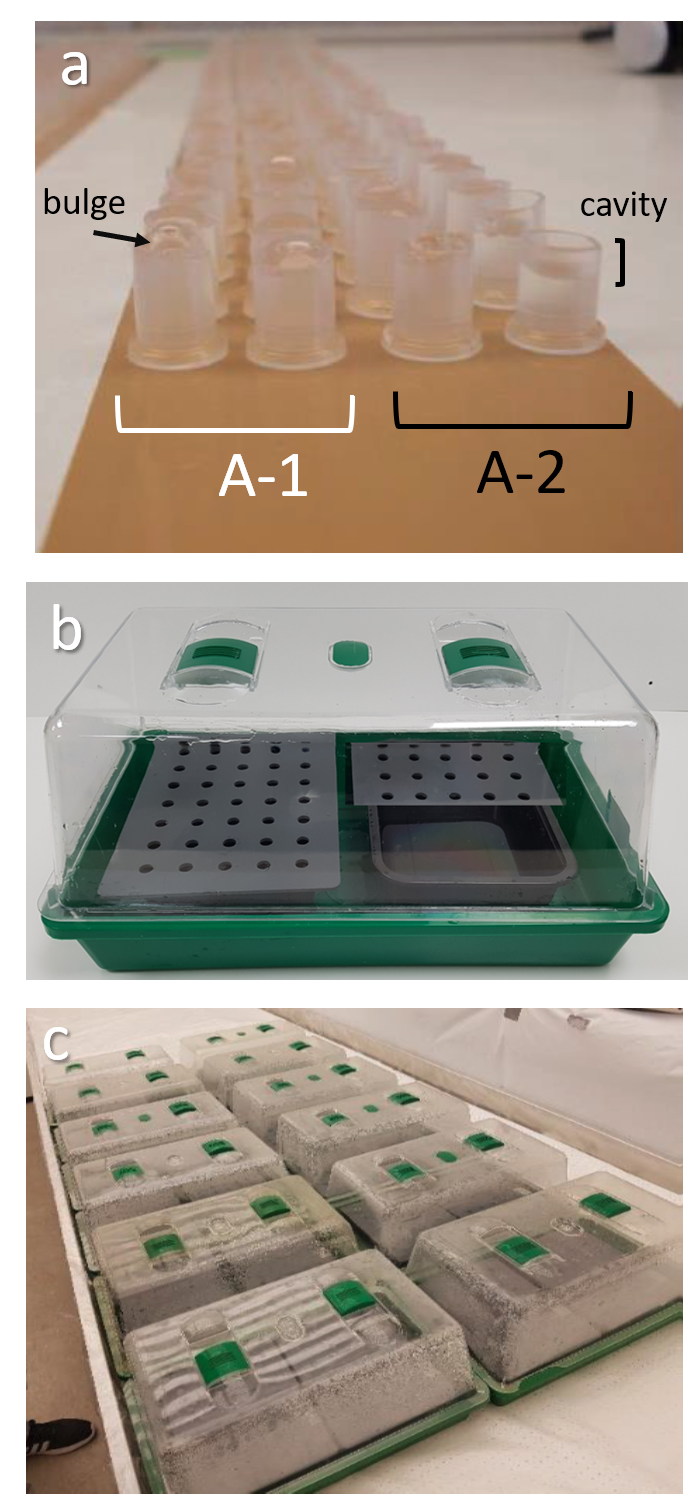


Fig. S12. Photos of germination system. (a) Microtubes filled with germination medium placed upside down on brown scotch adhesive tape. (b) A mini greenhouse with two 0.7 L PP (food grade) nutrient solution tanks (18.2 x 13.5 x 4.5 cm) covered by two BPA-free PVC lids (20 x 15 cm) containing 35 holes for microtubes. (c) Overview of germination phase in several mini greenhouses.
