# Supplementary figures and images for: Nutrient solutions for Arabidopsis thaliana: a study on nutrient solution composition in hydroponics systems
Source: Plant Methods. 2020 May 18;16:72. doi: 10.1186/s13007-020-00606-4 (PMC7324969; doi:10.1186/s13007-020-00606-4)

Additional file 13: Nutrient solution salt recipes


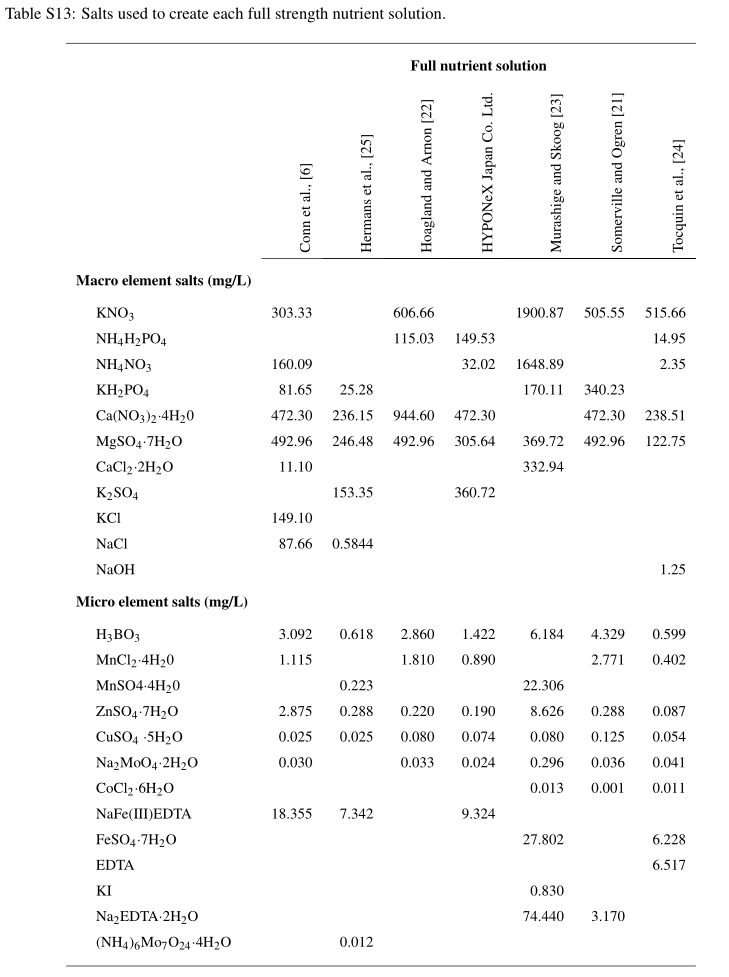

Supplement: Supplementary file 13 — Additional file 13. Chemical salts recipes used to prepare the full strenght nutrient solutions. [file 13007_2020_606_MOESM13_ESM.docx]
